# Synthesis and Systematic Investigation of Lepidiline A and Its Gold(I), Silver(I), and Copper(I) Complexes Using In Vitro Cancer Models and Multipotent Stem Cells

**DOI:** 10.1021/acsomega.4c05020

**Published:** 2024-07-15

**Authors:** Szilárd Tóth, Márton F. Szlávik, Réka Mandel, Fanni Fekecs, Gábor Tusnády, Flóra Vajda, Nóra Varga, Ágota Apáti, Attila Bényei, Attila Paczal, András Kotschy, Gergely Szakács

**Affiliations:** † Institute of Molecular Life Sciences, HUN-REN Research Centre for Natural Sciences, Magyar tudósok körútja 2, Budapest H-1117, Hungary; ‡ Servier Research Institute of Medicinal Chemistry, Záhony utca 7, Budapest H-1031, Hungary; § Hevesy György PhD School of Chemistry, Eötvös Loránd University, Pázmány Péter sétány 1/A, Budapest H-1117, Hungary; ∥ Doctoral School of Molecular Medicine, Semmelweis University, Budapest H-1089, Hungary; ⊥ Creative Cell Ltd., Puskas Tivadar u. 13, Budapest H-1119, Hungary; # Department of Physical Chemistry, University of Debrecen, Egyetem tér 1, Debrecen H-4032, Hungary; ∇ Center for Cancer Research, Medical University of Vienna, Spitalgasse 23, Vienna A-1090, Austria

## Abstract

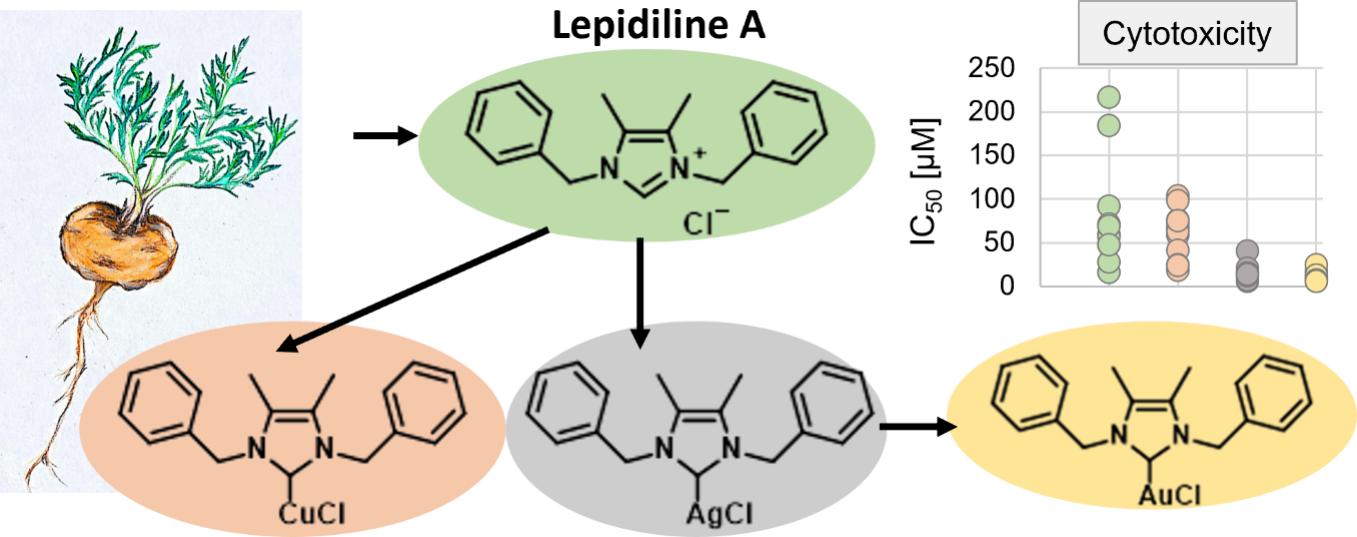

The imidazole alkaloid lepidiline A from the root of *Lepidium meyenii* has a moderate to low in vitro anticancer effect. Our aim was to extend cytotoxicity investigations against a panel of cancer cells, including multidrug-resistant cancer cells, and multipotent stem cells. Lepidiline A is a N-heterocyclic carbene precursor, therefore a suitable ligand source for metal complexes. Thus, we synthesized lepidiline A and its copper(I), gold(I), and silver(I) complexes and tested them against ovarian, gastrointestinal, breast, and uterine cancer cells and bone marrow-derived and adipose-derived mesenchymal stem cells. Lepidiline A and its copper complex demonstrated moderate cytotoxicity, while silver and gold complexes exhibited significantly enhanced and consistent cytotoxicity against both cancer and stem cell lines. ABCB1 in the multidrug-resistant uterine sarcoma line conferred significant resistance against lepidiline A and the copper-lepidiline A complex, but not against the silver and gold complexes. Our results indicate that only the copper complex induced a significant and universal increase in the production of reactive oxygen species within cells. In summary, binding of metal ions to lepidiline A results in enhanced cytotoxicity with the nature of the metal ion playing a critical role in determining its properties.

## Introduction

Lepidiline alkaloids are among the many active secondary plant metabolites that were identified by chemical analysis from *Lepidium meyenii*,^1−5^ also known as maca root, which is a nutritious Brassicaceae crop and herbal plant, domesticated in the Peruvian central Andes at least 3000 years ago.^6^

**Scheme 1:**
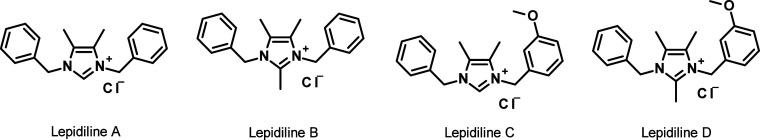
Structures of Lepidiline A, Lepidiline B, Lepidiline C, and Lepidiline D

Lepidilines are unique, putatively histidine-derived imidazole alkaloids,^2^ consisting of a few members only. Lepidilines A and B were first isolated around 20 years ago.^7^ More than 10 years later, their methoxy analogues lepidilines C and D were discovered (Scheme 1), and recently, additional variants, referred to as lepidilines E, F, and G, were found.^8−10^

Upon identification, lepidiline A and B were tested against a panel of cancer cell lines of various origins, and it was found that they exert only low or no toxicity up to 10 μg/mL (which is approximately 60 μM for lepidiline A).^7^ Lepidiline A and C were moderately toxic against the HL-60 leukemia line (IC_50_ values approximately 30 μM), while B and D were 1 order of magnitude more toxic (3.8 and 1.1 μM, respectively). In the case of the MCF-7 breast cancer line, only lepidiline C was active (75 μM), and none of the lepidilines were toxic against the HUVEC endothelial cell line up to 100 μM.^11^ Synthetic alkoxyamine derivatives and trifluoro derivatives of lepidilines elicited advanced cytotoxic effects,^12,13^ but these studies did not include the original plant lepidilines to allow a direct comparison.

Lepidilines A and C (but not B or D) can be considered as N-heterocyclic carbene (NHC) precursors. NHCs are potent electron donors to form complexes with almost every transition metal, which makes lepidilines A and C promising candidates for metal-based drug development. Lepidiline-metal complexes might elicit increased cytotoxicity, either as a complex or a carrier of toxic metals. A few iridium(I)-lepidiline complexes were synthesized, but their cytotoxicity is unknown.^14^ Nevertheless, numerous NHC-metal complexes, some of which were inspired by lepidilines but harboring extra moieties, were synthesized, and their biological activity was tested. Reports include Ru(II)- and Au(I)-N-heterocyclic carbenes,^15,16^ Ag(I)-NHCs, as antimicrobial and anticancer agents,^17,18^ and Cu(I)-NHCs possessing remarkable anticancer activity.^19^ Lepidiline-like platinum(II) complexes were also synthesized and were found to be active against cancer cells.^20,21^

However, metal complexes of lepidiline A or C were not investigated for their antiproliferative potential against cancer cells, and none of the plant-derived lepidilines were tested against multidrug-resistant (MDR) cancer or multipotent stem cells.

Therefore, our aim was to synthesize the natural compound lepidiline A (LA) and its metal complexes with the metals copper (Cu-LA), silver (Ag-LA), and gold (Au-LA) and to test their biological activity against a panel of cancer cell lines, including MDR phenotypes, and nonmalignant stem cell lines.

## Results and Discussion

### Synthesis and Characterization

**Scheme 2:**
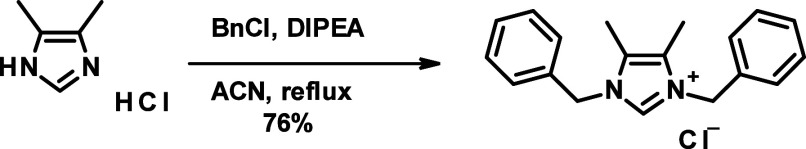
Synthesis of Lepidiline A Conditions: 3 equiv. of benzyl chloride (BnCl), 2 equiv. of *N*,*N*-diisopropylethylamine (DIPEA), 5 mL/mmol acetonitrile (ACN), 85 °C, yield: 76%.

In 2020, the first reported synthetic pathway for the synthesis of lepidiline A was documented.^14^ This synthetic route utilizes hydroxymethyl imidazolium salt as the initial starting material, which is subsequently reduced to 4,5-dimethyl imidazole. Similar to this route, 4,5-dimethyl-imidazole hydrochloride and benzyl chloride were employed for the synthesis of LA (Scheme 2). The resulting crude product was then subjected to reverse-phase preparative chromatography to achieve a high-purity product with a satisfactory yield (76%).

**Scheme 3:**
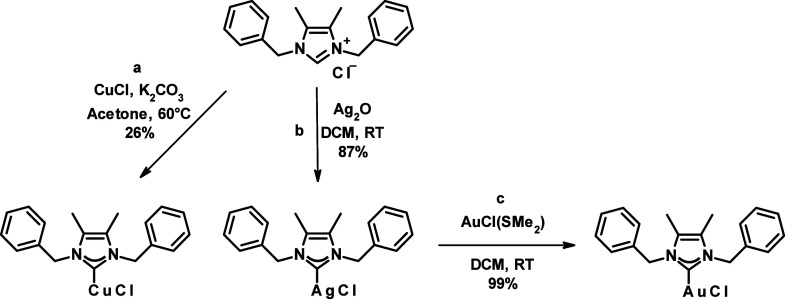
Synthesis of the Transition Metal Complexes Cu-LA, Ag-LA, and Au-LA from LA Conditions: (a) 1.1 equiv. of CuCl, 2 equiv. of K_2_CO_3_, 5 mL/mmol acetone, 60°C, yield: 26%; (b) 0.55 equiv. of Ag_2_O, 5 mL/mmol dichloromethane (DCM), room temperature (RT), yield: 87%; (c) 1.1 equiv. of AuCl(SMe_2_), 5 mL/mmol dichloromethane, room temperature, yield: 99%.

Lepidiline A was utilized as a starting material for the synthesis of copper, silver, and gold complexes employing two distinct methodologies (Scheme 3). The generation of the carbene under basic conditions in the presence of a metal source was employed for the synthesis of copper and silver complexes. Specifically, for the copper complex Cu-LA, a mixture of copper(I) chloride and K_2_CO_3_ was measured into a Schlenk vial under N_2_. Subsequently, degassed acetone was added through a septum, and the reaction mixture was heated to 60 °C and stirred for 1 h. The resulting mixture was then purified using flash chromatography on a silica column with eluents consisting of dichloromethane (DCM) and methanol (MeOH). The synthesis of Ag-LA required less stringent conditions. A mixture of LA and Ag_2_O was measured into a brown vial and dissolved in DCM under air. Notably, it is important to conduct this reaction in a dark environment to prevent the appearance of byproducts. The reaction proceeded to complete conversion within 30 min, after which the mixture was filtered, and Ag-LA was obtained by recrystallization utilizing diethyl ether. In the case of the gold complex synthesis, transmetalation from the silver complex was employed. This reaction occurred under mild conditions, resulting in excellent conversion and yield. Ag-LA and AuCl(SMe_2_) were dissolved in DCM and stirred for 1 h. The resulting mixture was then filtered, and Au-LA was subsequently crystallized using diethyl ether. The structures of the metal complexes were verified by ^1^H and ^13^C NMR spectroscopy, elemental analysis, and X-ray diffraction (Tables S3–S6 and Figures S2–S7). Measured by HPLC-UV, Cu-LA and Ag-LA were stable in a serum-free culture medium in the observed 48 h, while the signal for Au-LA decreased after 4 h (Table S7).

### In Vitro Cytotoxicity against Cancer Lines

**Figure 1:**
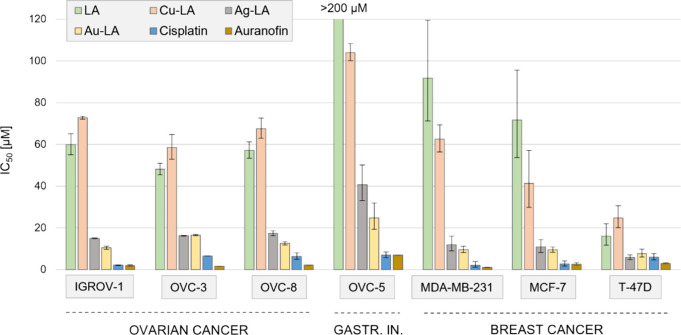
Cytotoxicity of LA, Cu-LA, Ag-LA, Au-LA, cisplatin, and auranofin against ovarium cancer lines, an upper gastrointestinal (gastr. in.) cancer line and breast cancer lines.

First, we tested the anticancer activity of LA and its metal complexes against 3 ovarium cancer lines (IGROV-1, OVC-3, and OVC-8) and compared it to the effect of cisplatin and auranofin. Cytotoxicities of LA and Cu-LA were moderate against the 3 cell lines (IC_50_ values between 48.1 and 72.9 μM), while Ag-LA and Au-LA were remarkably more cytotoxic (10.5–17.4 μM) but still less active than cisplatin (2.2–6.3 μM) or auranofin (1.7–2.3 μM) (Figure 1 and Table S1).

We tested the compounds also against OVC-5, a cell line originating from the upper gastrointestinal tract, that was misclassified earlier as an ovarian cancer line.^22^ Compared to the ovarian cancer lines, OVC-5 was 4 times less susceptible against LA (IC_50_ of 216.3 μM), 2.5-fold less toxic against Ag-LA (IC_50_ of 40.7 μM), and moderately less effective against Cu-LA and Au-LA (IC_50_ of 104.1 and 24.8 μM, respectively) and auranofin (7.0 μM), while sensitivity against cisplatin was in the same range (7.0 μM) (Figure 1 and Table S1).

Next, we tested the compounds against 3 breast cancer cell lines, including estrogen receptor (ER)-negative (MDA-MB-231) and ER-positive (MCF-7 and T-47D) lines. All cells were sensitive to cisplatin (2.2–6.0 μM) and auranofin (1.2–2.8 μM). Interestingly, T-47D was more sensitive to LA (IC_50_ of 16.1 μM), and to Cu-LA (IC_50_ of 24.8 μM), than the other two breast cancer lines, while Ag-LA and Au-LA were active against all 3 breast cancer cell lines (5.8–12.0 μM) (Figure 1 and Table S1). Although in a comprehensive study of plant alkaloids, lepidiline A was classified as a high-affinity ligand of human estrogen receptors α and β,^23^ susceptibility of T-47D seems to be independent of the ER status based on our cytotoxicity results.

In summary, LA and its metal derivatives were active against all cancer cells, especially against the T-47D breast cancer line. In the case of ovarian cancer lines and T-47D, activity increased in the order Cu-LA < LA ≪ Ag-LA ≤ Au-LA, while in the OVC-5 upper gastrointestinal line and MDA-MB-231 and MCF-7 breast cancer lines, the trend was LA < Cu-LA ≪ Ag-LA ≤ Au-LA. The cytotoxic effects were inferior to those of cisplatin and auranofin, except for T-47D, which was equally sensitive to Ag-LA, Au-LA, and cisplatin.

The increased sensitivity of the T-47D cell line is a novel finding. Growth of T-47D was shown to depend on the activity of HSD17B1,^24,25^ a protein that catalyzes estrogen activation by converting estrone (E1) to estradiol (E2). In theory, susceptibility of this line could be provoked by blocking the function of HSD17B1.^25,26^ Since LA was suggested to have a binding site on hamster HSD17B1,^27^ we performed a preliminary docking experiment, which predicted that LA accommodates in the steroid binding pocket of human HSD17B1 structures (Table S2 and Figure S1). Therefore, the LA binding to the HSD17B1 might cause the elevated sensibility of T-47D cells, but this hypothesis must be supported by further experiments, which is outside of the scope of this paper.

### Characterization of ABCB1-Mediated Multidrug Resistance

**Figure 2:**
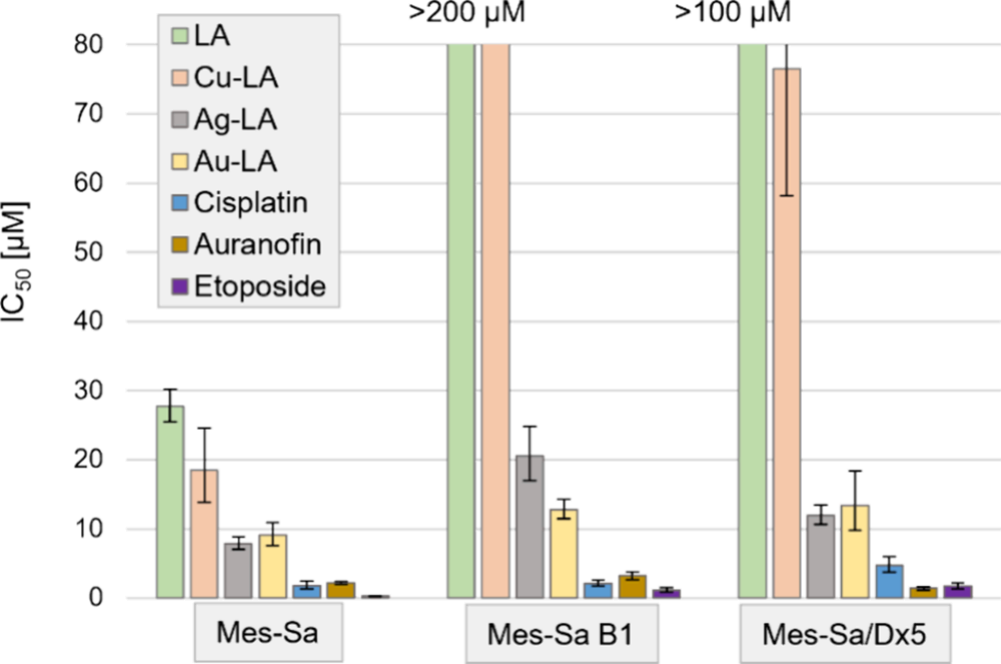
Cytotoxicity of LA, Cu-LA, Ag-LA, Au-LA, cisplatin, auranofin, and the ABCB1 substrate etoposide against the uterine sarcoma cell line Mes-Sa and its multidrug-resistant derivatives Mes-Sa B1 and Mes-Sa/Dx5.

Plant alkaloids are often recognized and eliminated by ABC transporters, as part of the process that protects the body from xenobiotics, the so-called “chemoimmunity” defense system,^28^ but these transporters can also cause cellular drug extrusion, leading to drug resistance, if expressed in cancer cells.^29,30^ Therefore, we tested the synthesized compounds against an in vitro multidrug-resistant (MDR) model system, consisting of the 3 uterine sarcoma cell lines Mes-Sa (parental line) and its ABCB1 expressing derivatives Mes-Sa B1 (MDR1 transfected) and Mes-Sa/Dx5 (doxorubicin selected). LA showed moderate cytotoxicity against Mes-Sa cells (27.8 μM), while it was not toxic against the MDR variants up to 200 μM. Similarly, Cu-LA exerted a moderate cytotoxicity against Mes-Sa (18.5 μM), while it was several-fold less active against both ABCB1 expressing cells. The Ag-LA and Au-LA complexes showed similar moderate cytotoxicity in all three cell lines (Figure 2 and Table S1), indicating their potential to overcome multidrug resistance.

### Formation of Reactive Oxygen Species

Numerous metal-based drugs, including imidazole-based NHC complexes, produce reactive oxygen species (ROS) through intracellular redox cycling.^31,32^ To get a glimpse of their action in the cells, we performed the H_2_DCFDA assay to detect intracellular ROS production of LA, Cu-LA, Ag-LA, Au-LA, and *tert*-butyl hydroperoxide (TBHP), a well-known inducer of mitochondrial ROS formation, in T-47D, MCF-7, and Mes-Sa cells.

**Figure 3:**
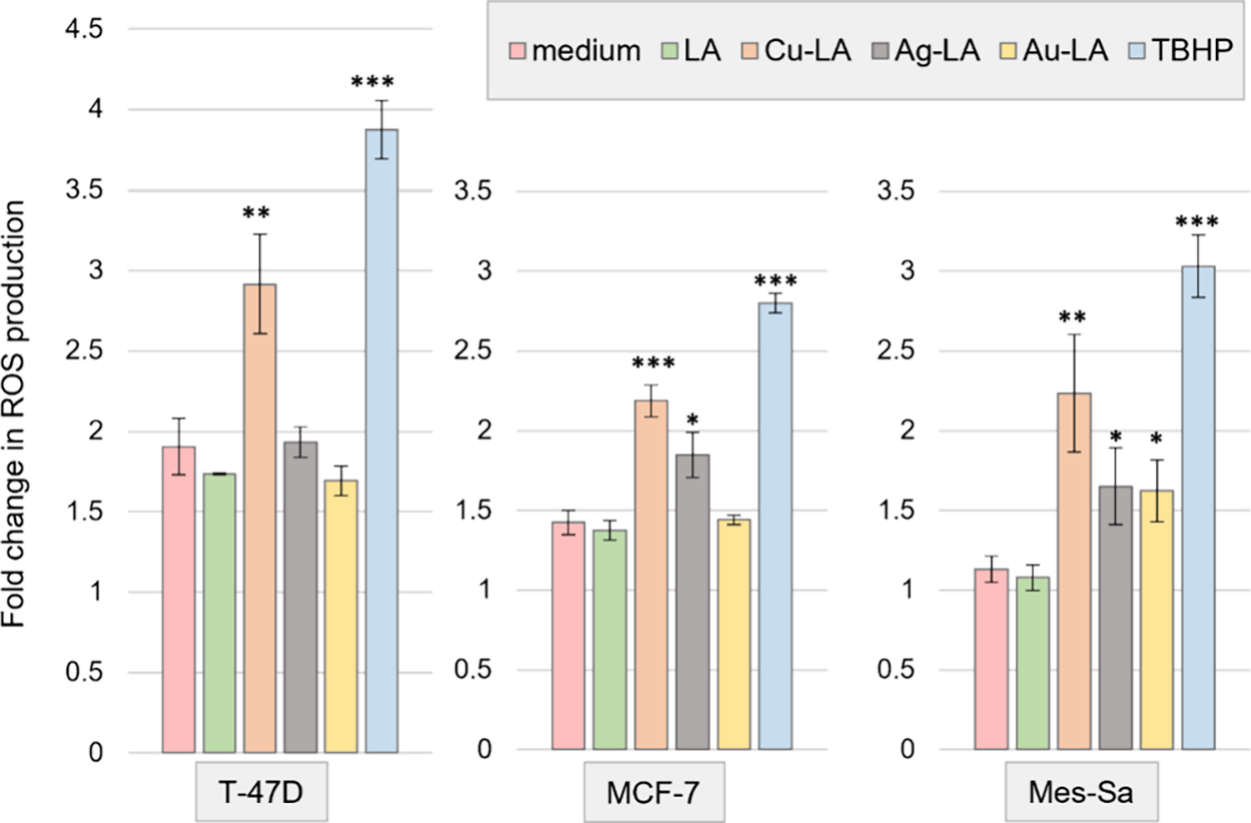
Intracellular ROS production assay by adding cell-permeable H_2_DCFDA to T-47D, MCF-7, and Mes-Sa and measuring its conversion to the highly fluorescent DCF after 4 h of incubation at 50 μM drug concentrations. TBHP: *tert*-butyl hydroperoxide. *p < 0.1; **p < 0.05; ***p < 0.01, compared to the medium.

During the 4 h of incubation, LA did not provoke extra ROS production in any of the cell lines compared to the medium control (Figure 3). On the contrary, Cu-LA induced an ∼2–3 fold higher intracellular ROS formation in all 3 cell lines. Ag-LA induced ∼50% more ROS in Mes-Sa and ∼30% more ROS in MCF-7 compared to the medium, but ROS was not elevated in T-47D. Au-LA induced ∼50% more ROS in Mes-Sa but not in the breast cancer lines.

The increased cytotoxicity of Ag-LA and Au-LA compared to LA is not related to ROS formation. In fact, other Ag- and Au-containing NHCs were reported to inhibit thioredoxin reductase.^17,18,33^ Meanwhile, Cu-LA, which was the least toxic metal-LA complex, induced intracellular ROS and might exert its cytotoxicity at least partly, through the production of these harmful radicals. Generally, the anticancer activity of copper complexes is attributed to the generation of intracellular reactive oxygen species by redox cycling between Cu(II) and Cu(I), especially with increased electron-withdrawing properties of the ligand.^34−36^

### Cytotoxicity against Mesenchymal Stem Cells

**Figure 4:**
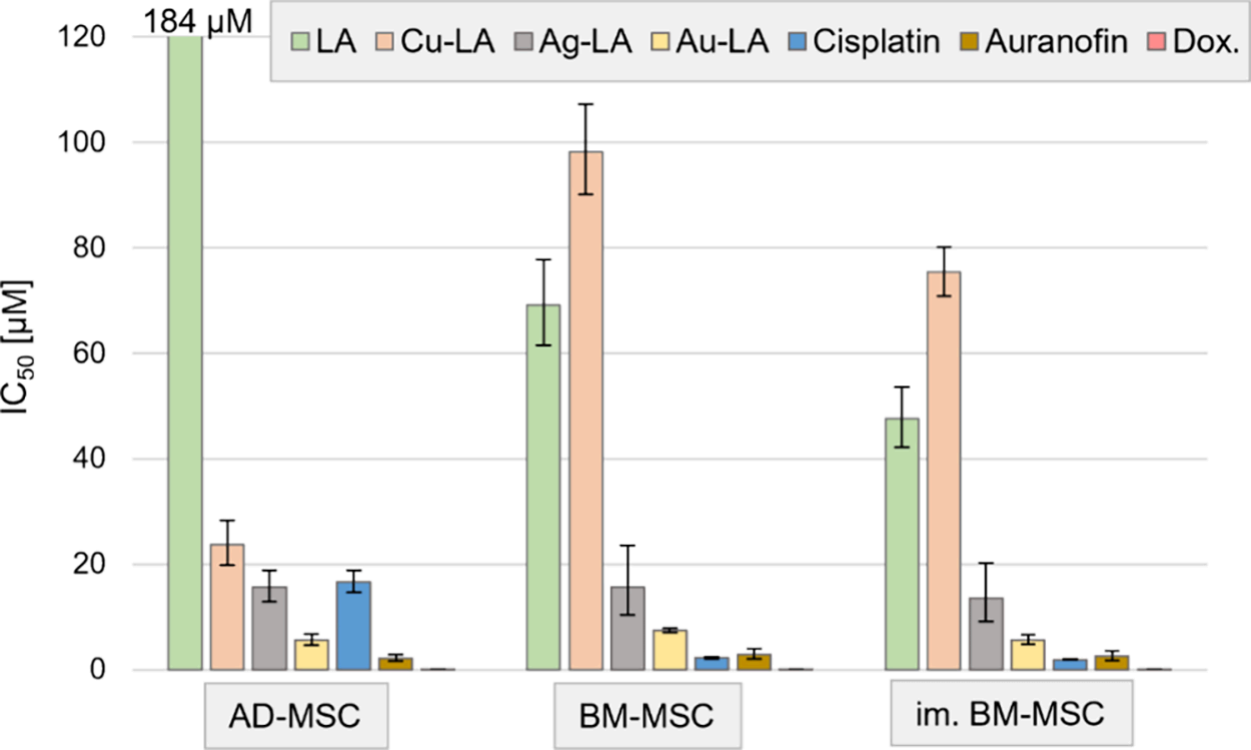
Cytotoxicity of LA and its copper, silver, and gold complexes (Cu-LA, Ag-LA, and Au-LA, respectively), cisplatin, auranofin, and doxorubicin (Dox) against adipose-derived MSC (AD-MSC), bone marrow-derived MSC (BM-MSC), and immortalized (im.) BM-MSC.

Cytotoxicity was also assessed against mesenchymal stem cell (MSC) lines, isolated from the adipose tissue and the bone marrow, and an immortalized bone marrow line^37^ (Figure 4). MSCs are multipotent stem cells, found in tissues, including fat, bone, and blood, with tissue regenerative capacity.^38^ In the tumor microenvironment, however, they can both suppress or support tumor growth by transforming into cancer-associated fibroblasts (CAFs).^39,40^

Lepidiline A was only slightly toxic (184 μM) against adipose-derived MSC (AD-MSC), while its metal complexes elicited cell killing at much lower concentrations (5.7–23.7 μM). Bone marrow MSCs (BM-MSC), on the contrary, were mildly sensitive against both LA and Cu-LA (48–98 μM) and were considerably more sensitive to Ag-LA and Au-LA (5.7–15.7 μM).

Cisplatin was slightly less toxic against AD-MSC (16.7 μM) and a bit more effective against BM-MSCs (2.0–2.3 μM) compared to cancer cells. Doxorubicin was equally cytotoxic against non-MDR cancer cells (0.034–0.22 μM) and MSCs (0.067–0.13 μM) (Figure 4 and Table S1).

The increased cytotoxicity of Cu-LA against AD-MSC might be linked to the elevated ROS production of Cu-LA, as AD-MSCs were more susceptible to H_2_O_2_ in vitro, compared to BM-MSCs.^41,42^ We found that MSCs had elevated susceptibility toward Au-LA (5.7–7.5 μM) compared to cancer lines (7.7–24.8 μM). There is a lack of data on how other gold(I)-NHCs act against MSCs; however, the cytotoxicity of auranofin, a gold(I)-containing potential anticancer drug candidate, which can inhibit myelopoiesis and induce eryptosis^43,44^ and can disrupt the thiol homeostasis in iPSCs,^45^ was similar in cancer cells (1.2–7.0 μM) and MSCs (2.2–2.9 μM); thus, there might be a general sensitivity of stem cells against gold(I)-NHCs, but not against all gold-containing compounds.

Overall, the cytotoxicity of LA and its metal complexes against MSCs might be beneficial in the tumor microenvironment, to control CAFs. For example, in bone metastasis, which occurs frequently in breast cancers, CAFs can support cancer cells by excreting various factors.^46^ Also, adipose-derived MSCs can be transformed to show a CAF-like tumor supporting phenotype by the conditioned medium from MCF-7 or MDA-MB-231 cultures, and this process plausibly happens also in breast cancer, where AD-MSCs are present in a high amount.^47^ Both cisplatin and doxorubicin are among the most used chemotherapeutics, and both were cytotoxic to MSCs, indicating that targeting cancer and CAFs at the same time might be a beneficial strategy. Meanwhile, effects of cytotoxic agents on stem cells can be associated with unwanted side effects as well. Thus, to investigate, if the action of lepidilines against MSCs can be exploited against the tumor supporting CAFs, a further comprehensive study is needed that is outside of the scope of this paper.

In conclusion, the prevalence of metal-containing anticancer agents is increasing in drug research, with the vision that they possess potential to overcome platinum drug resistance. NHCs, especially imidazolium-NHCs complexed with gold, silver, and ruthenium, gained attention due to their versatility.^33,48−50^ In this work, we demonstrated that the imidazolium salt lepidiline A and its metal complexes Cu-LA, Ag-LA, and Au-LA exhibited anticancer cytotoxicity against a panel of cancer cells, particularly against the breast cancer line T-47D, and also against adipose- and bone marrow-derived stem cells. LA and Cu-LA were in general less active, while Ag-LA and Au-LA were more cytotoxic. The marginal stem cell targeting trait of Au-LA raises concerns for Au-based NHCs, while Ag-LA was equipotent against cancer and stem cells.

## Experimental Section

### General Experimental Methods

The starting materials and reagents, unless otherwise indicated, are commercially available and were used without further purification. The reactions were monitored by using GC-MS, HPLC-MS, HPLC-UV, TLC, and NMR measurements.

For the GC-MS tests, an Agilent 6850 gas chromatograph (15 m × 0.25 mm column, 0.25 mm HP-5MS coating, He carrier gas) and an Agilent 5975C mass spectrometer (ion source: EI+, 70 eV, 230 °C, quadrupole: 150 °C, interface: 300 °C) were used. The HPLC-UV tests were performed with an Agilent Technologies 1200-type liquid chromatograph, and the HPLC-MS tests were performed with the chromatograph connected to a quadrupole mass analyzer and a combined APCI/ESI ion source, with which we were able to measure both in negative and positive modes. Eluent A: water, 5% acetonitrile, and 0.05% TFA; eluent B: acetonitrile, 5% water, and 0.075% TFA. The device also had a DAD 190–400 nm detector, with which we detected at 210 and 254 nm. The NMR spectroscopy tests were performed on a Bruker UltraShield Plus 500 and a Bruker UltraShield Plus 400 equipped with an automatic sample changer. CDCl_3_ and DMSO-*d*_6_ were used as solvents. Chemical shifts (δ) were given in ppm using solvent signals as internal standards; CDCl_3_ (^1^H δ 7.26 ppm, ^13^C δ 77.16 ppm) and DMSO-*d*_6_ (^1^H δ 2.50 ppm, ^13^C δ 39.52 ppm). Coupling constants (*J*) were given in Hertz (Hz). The following was used to denote the splits: s (singlet), d (doublet), t (triplet), q (quintet), sp (septet), m (multiplet), br s (broad singlet), br d (broad doublet), dd (doublet of doublet), td (doublet of triplet), dt (triplet of doublet), and tm (multiplet of triplet).

The normal-phase column chromatographic separations were performed using a Teledyne Isco CombiFlash Rf-type flash chromatographic device, using Teledyne Isco RediSep-type columns filled with normal-phase silica. The reversed-phase column chromatographic separations were performed using a Teledyne Isco EZ Prep UV-type flash chromatographic device, using Teledyne Isco RediSep Rf-type columns with C18 filling.

The HR-MS measurements were performed with an Agilent Technologies 6230 TOF LC/MS mass analyzer with an ESI ion source connected to an Agilent Technologies 1200 liquid chromatograph. In the case of the complexes, Cu-LA, Ag-LA, and Au-LA HR-MS could not be measured.

#### Lepidiline A (LA, 1,3-Dibenzyl-4,5-dimethyl-imidazol-1-ium-chloride)

For the synthesis of LA, 1.00 g (7.54 mmol) of 4,5-dimethyl-imidazol-3-ium-chloride was measured into a round-bottom flask equipped with a magnetic stirring bar; then, it was dissolved in 37.7 mL of acetonitrile. To the mixture, 2.6 mL (22.60 mmol) of benzyl chloride and 2.6 mL (15.10 mmol) of diisopropyl-ethyl-amine were measured, and the mixture was heated up and stirred for 16 h at 85 °C. To the mixture, Celite was added, and all the volatiles were removed under pressure; then, the mixture was purified via reversed-phase chromatography using water and acetonitrile. All the solvents were lyophilized to get LA as a white solid. Yield = 1.78 g (5.70 mmol, 75.6%).

^1^H NMR (500 MHz, DMSO-*d*_6_): δ ppm 9.34 (s, 1 H, *2*), 7.47–7.28 (m, 10 H, *8–12 + 15–19*), 5.44 (s, 4 H, *6 + 13*), 2.12 (s, 6 H, *20 + 21*). ^13^C NMR (125 MHz, DMSO-*d*_6_): δ ppm 135.9 (*2*), 134.7 (*7 + 14*), 129.6 (*10 + 17*), 129.0 (*9 + 11 + 16 + 18*), 128.2 (*8 + 12 + 15 + 19*), 127.6 (*4 + 5*), 50.1 (*6 + 13*), 8.6 (*20 + 21*) (Figure S2). 2HRMS (ESI) M+ found = 277.1700 (δ = 0.3 ppm).

#### Cu-LA (Chloro-(1,3-dibenzyl-4,5-dimethyl-imidazol-2-ylidene)copper)

For the synthesis of Cu-LA, 100.0 mg (0.32 mmol) of LA, 34.8 mg (0.35 mmol) of CuCl, and 88.4 mg (0.64 mmol) of K_2_CO_3_ were measured into a Schlenk vial equipped with a magnetic stirring bar, then inerted with N_2_ gas, and sealed with a septum. Through the septum, 1.6 mL of acetone was added, and the mixture was heated up and stirred for 1 h at 60 °C. To the mixture, Celite was added, and all the volatiles were removed under reduced pressure; then, it was purified via normal-phase chromatography using dichloromethane and methanol as eluents. All the volatiles were removed to get Cu-LA as a greenish white solid. Yield: 30.0 mg (0.08 mmol, 25.00%).

^1^H NMR (400 MHz, CDCl_3_): δ ppm 7.45–7.36 (m, 4 H, *9 + 11 + 16 + 18*), 7.36–7.30 (m, 2 H, *10 + 17*), 7.22 (d, *J* = 7.38 Hz, 4 H, *8 + 12 + 15 + 19*), 5.38 (s, 4 H, *6 + 13*), 1.99 (s, 6 H, *20 + 21*). ^13^C NMR (100 MHz, CDCl_3_) δ ppm 135.8 (*7 + 14*), 129.0 (*9 + 11 + 16 + 18*), 128.2 (*10 + 17*), 126.9 (8 + 12 + 15 + 19), 126.9 (*4 + 5*), 53.0 (*6 + 13*), 9.3 (*20 + 21*) (Figure S3).

#### Ag-LA (Chloro-(1,3-dibenzyl-4,5-dimethyl-imidazol-2-ylidene)silver)

For the synthesis of Ag-LA, 500.0 mg (1.60 mmol) of LA was measured into a brown vial equipped with a magnetic stirring bar, and it was dissolved in 8.0 mL of dichloromethane. Ag_2_O (203.7 mg, 0.88 mmol) was added, and the mixture was stirred at room temperature for 30 min. The mixture was filtered and concentrated under reduced pressure. Ag-LA, a white solid, was then crystallized with the addition of diethyl ether. Yield: 586.0 mg (1.40 mmol, 87.35%).

^1^H NMR (500 MHz, CDCl_3_): δ ppm 7.39–7.28 (m, 6 H, *9 + 10 + 11 + 16 + 17 + 18*), 7.13 (d, *J* = 7.40 Hz, 4 H, *8 + 12 + 15 + 19*), 5.31 (s, 4 H, *6 + 13*), 1.98 (s, 6 H, *20 + 21*). ^13^C NMR (125 MHz, CDCl_3_): δ ppm 178.8 (*2*), 135.6 (*7 + 14*), 129.1/128.3 (*9 +10 + 11 + 16 + 17 + 18*), 126.6 (*8 + 12 + 15 + 19*), 126.4 (*4 + 5*), 53.5* (*6 + 13*), 9.4 (*20 + 21*) (Figure S4).

#### Au-LA (Chloro-(1,3-dibenzyl-4,5-dimethyl-imidazol-2-ylidene)gold)

For the synthesis of Au-LA, 100.0 mg (0.24 mmol) of Ag-LA was measured into a brown vial equipped with a magnetic stirring bar and dissolved in 1.2 mL of dichloromethane; then, 77.2 mg (0.26 mmol) of AuCl(SMe_2_) was added, and the mixture was stirred at room temperature for 1 h. The mixture was filtered, and the volatiles were concentrated under reduced pressure; then, Au-LA, a white solid, was crystallized with the addition of diethyl ether. Yield: 120.0 mg (0.24 mmol, 98.99%).

^1^H NMR (400 MHz, CDCl_3_): δ ppm 7.39–7.20 (m, 10 H, *8–18 + 15–19*), 5.44 (s, 4 H, *6 + 13*), 1.98 (s, 6 H, *20 + 21*). ^13^C NMR (100 MHz, CDCl_3_): δ ppm 170.2 (*2*), 135.3 (*7 + 14*), 129.0 (*10 + 17*), 128.3 (*9 + 11 + 16 + 18*), 126.9 (*8 + 12 + 15 + 19*), 125.8 (*4 + 5*), 52.7 (*6 + 13*), 9.4 (*21 + 21*) (Figure S5).

### Characterization of Metal Complexes

Elemental analytic measurements of Cu-LA, Ag-LA, and Au-LA were carried out using a Thermo Fisher Flash EA 1112 Series CHNS-O analyzer, with the oven temperature of 950 °C. The results of the measurements showed close matching with the expected atomic ratios (Tables S3–S5). The solid-state structures of metal complexes were verified by single-crystal X-ray diffraction. The powders were dissolved in DCM followed by slow solvent evaporation and crystal formation. Crystals from Ag-LA and Au-LA (but not from Cu-LA) were suitable for testing. Ag-LA and Au-LA formed dimers, and the structures were confirmed (Table S6 and Figures S6 and S7).

Stability of the metal complexes in solution was measured in serum-free culture medium. The compounds were dissolved in DMSO right before the experiment. Solutions of Cu-LA and Ag-LA contained 1% final volume of DMSO, while Au-LA contained 30% DMSO. Samples were taken right after dilution, and after 1 h, 2 h, 4 h, 8 h, 16 h, 32 and 48 h, and measured by HPLC-UV.

### Cell Lines and Culture Conditions

OVCAR-3 (OVC-3) and OVCAR-8 (OVC-8) ovarian serous adenocarcinomas, IGROV-1 ovarian endometrioid adenocarcinoma, OVCAR-5 (OVC-5) upper gastrointestinal carcinoma, MDA-MB-231 breast adenocarcinoma, and MCF-7 and T-47D invasive breast carcinomas were purchased from the Developmental Therapeutic Program of the National Cancer Institute (NIH, USA) and were cultured in an RPMI medium (Thermo Fisher). Mes-Sa and Mes-Sa/Dx5 uterine sarcoma cell lines were purchased from American Type Culture Collection (ATCC) and were kept in DMEM (Thermo Fisher). Mes-Sa B1 was transfected with the human MDR1 gene, and Mes-Sa mCherry, Mes-Sa B1 mOrange, and Mes-Sa/Dx5 eGFP were engineered to stably express the respective fluorescent proteins previously.^51^ Culture media for cancer cells were supplemented with 10% fetal bovine serum, 2 mM l-glutamine, and 100 units/mL penicillin and 100 μg/mL streptomycin (Thermo Fisher). The adipose tissue and bone marrow-derived mesenchymal stem cells (AD-MSC and BM-MSC) were described previously^37^ and were kept in DMEM-F12 (Gibco) supplemented with 10% fetal bovine serum, l-glutamine (Thermo Fisher), 0.1% gentamicin (Gibco, 50 mg/mL), and 16 ng/mL fibroblast growth factor 2 (Peprotech). Cells were kept at 37 °C under 5% CO_2_ and were negative for mycoplasma infection.

### Cytotoxicity Tests

Cytotoxicities of the nonfluorescent cancer cells were assessed with a PrestoBlue cell viability reagent (Thermo Fisher). Briefly, 2500 cells/well were seeded on 384-well plates; then, the next-day drugs were added. After 72 h of incubation, PrestoBlue was added in a 10% final concentration, and after an hour of additional incubation, plates were measured with a PerkinElmer EnSpire plate reader at 555 nm/585 nm excitation/emission wavelengths. Stem cells were seeded on 96-well plates at a density of 5000 cells/well, and the following day, drugs were added for 96 h of incubation; then, viability was assessed with 10% PrestoBlue diluted in PBS. Fluorescent cancer cells were admixed before seeding on 384-well plates at an 800 cells/well density each, thus altogether 2400 cells/well. Drugs were added the following day, and the intensity of the fluorescent proteins was measured after 144 h of incubation at the respective fluorescent channels (excitation/emission, eGFP: 485 nm/510 nm; mCherry: 585 nm/610 nm; mOrange: 545 nm/567 nm). pIC_50_ values were calculated by a custom program written by Judit Sessler in C#. IC_50_ values and standard deviation were calculated from average pIC_50_ values from at least 3 independent measurements.

### Molecular Docking

Ligand-free, E1, and E2 bounded structures of HSD17B1 were downloaded from the PDB database^52^ (PDB codes 1BHS, 1QYV, and 1A27, respectively). Atomic coordinates of **LA** were downloaded from the PubChem database^53^ in SDF format. For docking, the Pymol plugin of Autodock Vina (v1.2.3)^54^ was used with default parameters to generate poses with the 100 lowest energies.

### H_2_DCFDA Tests

To assess intracellular ROS production, we used a H_2_DCFDA cellular ROS assay kit (ab113851, Abcam) according to the manufacturer’s instructions. Briefly, 20,000 cells were seeded on 96-well plates. On the following day, cells were washed, and H_2_DCFDA solution, prepared by avoiding direct light, was added for 45 min. Next, cells were washed, and 50 μM samples of test compounds were aspirated on the cells in a complete and phenol red-free medium (FluoroBrite, Thermo Fisher). At each hour, fluorescence of 2′,7′-dichlorofluorescein (DCF) was measured at 484 nm/535 nm excitation/emission wavelengths. Values were normalized to 0 h measurements. Values indicate at least 3 independent measurements.

## Supporting Information Available

Crystallographic information on Ag-LA and Au-LA (CIF)
Cytotoxicity (IC_50_ values); prediction of binding energy release; LA and estradiol docking in HSD17B1; ^1^H and ^13^C NMR spectra figures, results of elemental analysis, and X-ray diffraction study (deposition number for supplementary crystallographic data for Ag-LA: 2356399, for Au-LA: 2356400, provided free of charge by the joint Cambridge Crystallographic Data Centre and Fachinformationszentrum Karlsruhe Access Structures service); stability of the metal complexes in solution (PDF)

## Supplementary Material

ao4c05020_si_001.cif
