# Supplementary material for: Synthesis and Systematic Investigation of Lepidiline A and Its Gold(I), Silver(I), and Copper(I) Complexes Using In Vitro Cancer Models and Multipotent Stem Cells
Source: ACS Omega. 2024 Jul 15;9(29):32226–34. doi: 10.1021/acsomega.4c05020 (PMC11270681; doi:10.1021/acsomega.4c05020)
Supplement: Supplementary file 2 — ao4c05020_si_002.pdf [file ao4c05020_si_002.pdf]

Supporting information

for

Synthesis and systematic investigation of Lepidiline A and its gold(I), silver(I) and copper(I) complexes using in vitro cancer models and multipotent stem cells

Szilárd Tóth[a§*], Márton F. Szlávik[b,c§], Réka Mandel[a], Fanni Fekecs[b], Gábor Tusnády[a], Flóra Vajda[a,d], Nóra Varga[a,e], Ágota Apáti[a], Attila Bényei[f], Attila Paczal[b], András Kotschy[b], Gergely Szakács[a,g]

[a]Institute of Molecular Life Sciences, HUN-REN Research Centre for Natural Sciences, Magyar tudósok körútja 2, H-1117, Budapest, Hungary

[b]Servier Research Institute of Medicinal Chemistry, Záhony utca 7, H-1031, Budapest, Hungary

[c]Hevesy György PhD School of Chemistry, Eötvös Loránd University, Pázmány Péter sétány 1/A, H-1117, Budapest, Hungary

[d]Doctoral School of Molecular Medicine, Semmelweis University, H-1089, Budapest, Hungary

[e]Creative Cell Ltd, Puskás Tivadar u. 13, H-1119, Budapest, Hungary

[f]University of Debrecen, Department of Physical Chemistry, Egyetem tér 1, H-4032, Debrecen, Hungary

[g]Center for Cancer Research, Medical University of Vienna, Spitalgasse 23, A-1090, Vienna, Austria

*Email: toth.szilard.enzim@ttk.hu; Phone: +36 1 3826 737;

[§]S. Tóth and M.F. Szlávik contributed equally.

Table of contents

Table S1. Cytotoxicity of the tested compounds

Table S2. Binding energy release

Table S3-S5. Elemental analysis of Cu-LA, Ag-LA and Au-LA

Table S6. Experimental details of X-ray diffraction study

Table S7. Stability in solution of Cu-LA, Ag-LA and Au-LA

Figure S1. Docking of estrone and lepidiline A

Figure S2-S5. ^{1}H and ^{13}C NMR Spectra

Figure S6. X-ray structures and geometric parameters of Ag-LA

Figure S7. X-ray structures and geometric parameters of Au-LA

Table S1. Cytotoxicity of the tested compounds

IC_{50} values and the respective standard deviations (SD) calculated from pIC_{50} values for all the tested compounds against ovarian cancer cell lines (IGROV-1, OVC-3, OVC-8), an upper gastrointestinal cancer line (OVC-5), breast cancer lines (MDA-MB-231, MCF-7, T-47D), uterine sarcoma lines (Mes-Sa, Mes-Sa/B1, Mes-Sa/Dx5), adipose tissue derived multipotent stem cell line (AD-MSC), bone-derived multipotent cell lines (BM-MSC, immortalized BM-MSC). LA: lepidiline A, Cu-LA: copper(I)-lepidiline A, Ag-LA: silver(I)-lepidiline A, Au-LA: gold(I)-lepidiline A.

	**LA**		**Cu-LA**		**Ag-LA**		**Au-LA**		**Cisplatin**		**Auranofin**		**Doxorubicin**		**Etoposide**	
**Cell line**	**IC_{50}**	+SD / -SD	**IC_{50}**	+SD / -SD	**IC_{50}**	+SD / -SD	**IC_{50}**	+SD / -SD	**IC_{50}**	+SD / -SD	**IC_{50}**	+SD / -SD	**IC_{50}**	+SD / -SD	**IC_{50}**	+SD / -SD
IGROV-1	**59.9**	5.18/4.77	**72.9**	0.67/0.67	**15.0**	0.17/0.17	**10.5**	0.77/0.72	**2.19**	0.17/0.16	**1.93**	0.45/0.36	**-**		**-**	
OVC-3	**48.1**	2.85/2.69	**58.5**	6.24/5.64	**16.3**	0.15/0.15	**16.6**	0.35/0.34	**6.64**	0.06/0.06	**1.71**	0.08/0.07	**-**		**-**	
OVC-8	**57.2**	4.09/3.82	**67.6**	5.00/4.66	**17.4**	1.20/1.12	**12.7**	0.84/0.79	**6.34**	1.79/1.40	**2.27**	0.02/0.02	**0.19**	0.07/0.05	**-**	
OVC-5	***>200***		**104.1**	4.16/4.00	**40.7**	9.50/7.70	**24.8**	7.01/5.47	**7.00**	1.51/1.24	**6.96**	0.08/0.08	**-**		**-**	
MDA-MB-231	**91.8**	27.8/20.6	**62.5**	6.83/6.15	**12.0**	3.96/2.97	**9.58**	1.62/1.39	**2.17**	1.83/0.99	**1.15**	0.14/0.12	**0.22**	0.07/0.06	**-**	
MCF-7	**71.7**	23.9/17.9	**41.3**	15.7/11.4	**10.9**	3.53/2.66	**9.49**	1.36/1.19	**2.71**	1.53/0.98	**2.83**	0.88/0.67	**-**		**-**	
T-47D	**16.1**	5.97/4.35	**24.8**	5.76/4.38	**5.83**	1.26/1.04	**7.71**	2.11/1.66	**5.99**	1.72/1.33	**2.31**	1.15/0.77	**-**		**-**	
Mes-Sa	**27.8**	2.47/2.27	**18.5**	6.10/4.58	**7.90**	0.94/0.84	**9.09**	1.79/1.49	**1.84**	0.67/0.49	**2.21**	0.18/0.17	**0.038**	0.01/0.01	**0.26**	0.07/0.06
Mes-Sa/B1	***>200***		***>100***		**20.5**	4.32/3.57	**12.8**	1.47/1.32	**2.09**	0.50/0.41	**3.22**	0.61/0.51	**-**		**1.17**	0.33/0.26
Mes-Sa/Dx5	***>200***		**76.6**	24.1/18.3	**12.0**	1.49/1.32	**13.4**	5.01/3.64	**4.71**	1.29/1.01	**1.40**	0.26/0.22	**2.41**	0.97/0.69	**1.70**	0.47/0.37
AD-MSC	**184.3**	16.2/14.9	**23.7**	4.64/3.88	**15.6**	3.31/2.73	**5.69**	1.07/0.90	**16.7**	2.24/1.97	**2.16**	0.76/0.56	**0.13**	0.03/0.02	-	
BM-MSC	**69.1**	8.6/7.6	**98.3**	8.9/8.2	**15.7**	8.01/5.03	**7.54**	0.45/0.42	**2.32**	0.23/0.21	**2.88**	1.21/0.85	**0.10**	0.01/0.01	-	
im. BM-MSC	**47.6**	6.1/5.4	**75.4**	4.8/4.5	**13.6**	6.65/4.47	**5.70**	0.97/0.83	**1.97**	0.09/0.09	**2.61**	1.06/0.75	**0.067**	0.01/0.01	-	

Table S2.

Binding energy release (ΔG) by docking NADPH, estrone, estradiol and LA to HSD17B1 structures, calculated with AutoDock Vina (v1.2.3) [1]. The conversion of E1 to E2 by HSD17B1 starts with NADPH binding to the cofactor binding site (COF), with consecutive conformational change that opens the substrate binding pocket (SUB), followed by E1 binding. When the hydride transfer takes place, at first, NADP+ leaves, then E2 exits [2]. In a cofactor and substrate free structure (1BHS), COF accommodates NADPH with high affinity (ΔG: -11.5 kcal/mol). Although physiologically SUB is occluded in the apoenzyme until NADPH binds, LA, E1 and E2 were docked in SUB instead of COF. In a crystal structure harboring only NADP (1QYV), when SUB is physiologically available, E1, E2 and LA were all docked to SUB. The predicted free energy release by the binding of estrogens triggered 9.1 kcal/mol and 9.0 kcal/mol for E1 and E2, respectively, which is an order of magnitude greater than the free energy release upon LA binding (-8.2 kcal/mol). After we removed E2 from a crystal structure containing both NADPH and E2 (1A27), both E1 and E2 docked in SUB with high affinity (-9.2 kcal/mol and -9.4 kcal/mol, respectively), while LA docked with -7.5 kcal/mol).

Although, compared to E1 and E2 docking, the estimated binding energy release was significantly lower in case of LA docking, since LA's concentration is approximately 3-order of magnitude higher at cytotoxic concentrations compared to estrogens [3], it might occupy the binding pockets, causing HSD17B1 inhibition, as binding energy release is proportional to the logarithm of concentration ($\Delta G = -RT \ln(c)$).

			**Calculated ΔG**			
**PDB ID**	**COF**	**SUB**	**NADPH to COF**	**Estrone to SUB**	**Estradiol to SUB**	**LA to SUB**
1BHS	-	-	-11.5	-8.1	-8.9	-7.9
1QYV	NADPH	-	-	-9.1	-9.0	-8.2
1A27	NADPH	(E2 removed)	-	-9.2	-9.4	-7.5

References

1. Morris, G.M., et al., AutoDock4 and AutoDockTools4: Automated docking with selective receptor flexibility. J Comput Chem, 2009. **30**(16): p. 2785-91.
2. Negri, M., M. Recanatini, and R.W. Hartmann, Insights in 17beta-HSD1 enzyme kinetics and ligand binding by dynamic motion investigation. PLoS One, 2010. **5**(8): p. e12026.
3. Zhang, C.Y., et al., The contribution of 17beta-hydroxysteroid dehydrogenase type 1 to the estradiol-estrone ratio in estrogen-sensitive breast cancer cells. PLoS One, 2012. **7**(1): p. e29835.

Table S3: Results of the elemental analytic measurements for Cu-LA

**Molecular Data**		**Structure**
Type	**Principal**	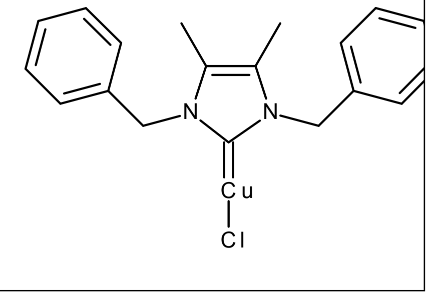
Theoretical	**1**	
Molecular Weight	**375.37**	
Exact Mass	**374.0611**	
Formula	**C19 H20 Cl Cu N2**	

Element	Theory	Results		Mean	Deviation
**C**	60.79	59.79	60.02	59.91	-0.89
**H**	5.37	5.22	5.34	5.28	-0.09
**N**	7.46	7.04	7.06	7.05	-0.41
**Cl**	9.44				
**Cu**	16.93				

Table S4: Results of the elemental analytic measurements for Ag-LA

Molecular Data		Structure
Type	**Principal**	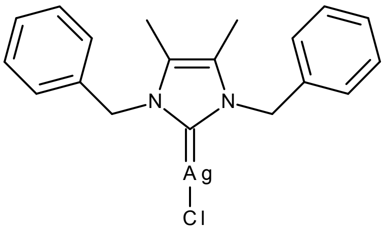
Theoretical	**1**	
Molecular Weight	**419.7**	
Exact Mass	**418.0366**	
Formula	**C19 H20 Ag Cl N2**	

Element	Theory	Results		Mean	Deviation
**C**	54.37	54.69	54.74	54.72	0.34
**H**	4.80	4.90	4.87	4.89	0.08
**N**	6.67	6.45	6.40	6.43	-0.25
**Ag**	25.70				
**Cl**	8.45				

Table S5: Results of the elemental analytic measurements for Au-LA

Molecular Data		Structure
Type	**Principal**	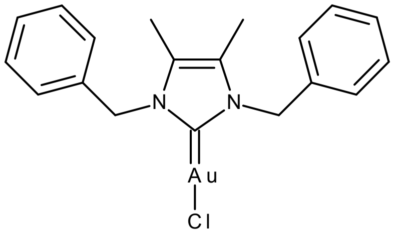
Theoretical	**1**	
Molecular Weight	**508.8**	
Exact Mass	**508.0981**	
Formula	**C19 H20 Au Cl N2**	

Element	Theory	Results		Mean	Deviation
**C**	44.85	43.06	42.91	42.99	-1.87
**H**	3.96	3.62	3.85	3.74	-0.23
**N**	5.51	5.06	5.07	5.07	-0.44
**S**		1.14	0.24	0.69	
**Au**	38.71				
**Cl**	6.97				

Table S6. Experimental details of X-ray diffraction of Ag-LA and Au-LA

Suitable crystals were fixed on a Mitegen loop using high viscosity oil. Data were collected at room temperature using a Bruker-D8 Venture diffractometer equipped with INCOATEC IμS 3.0 dual (Cu and Mo) sealed tube microsources and Photon 200 Charge-integrating Pixel Array detector, MoKα (λ = 0.71073 Å) radiation was used. Data collection and integration was performed using the APEX4 software [4]. Data reduction and multi-scan absorption correction was applied [5]. The structure could be easily solved using direct methods and refined on F^2 using SHELXL program [6] incorporated into the APEX4 suite. Refinement was performed anisotropically for non-hydrogen atoms except two phenyl carbon atoms in Au-LA as turning them anisotropically they became non positive definit. Hydrogen atoms were placed into geometric positions. Tables were extracted from the edited CIF file using publCIF [7]. The PLATON program was used for crystallographic calculations.

References:

4. APEX4 v2017.3-0, Bruker AXS.
5. SAINT V8.38A, Bruker AXS Inc., 2017. SADABS2016/2 - Bruker AXS area detector scaling and absorption correction
6. SHELXT 2014/5 (Sheldrick, 2014); program(s) used to refine structure: SHELXL2016/6 (Sheldrick, 2016).
7. publCIF: Westrip, S. P. (2010). J. Appl. Cryst. 43, 920–925.

Compound	Ag-LA	Au-LA
Crystal data		
Chemical formula	$C_{19}H_{20}AgClN_2$	$C_{19}H_{20}AuClN_2$
M_r	419.69	508.79
Crystal system, space group	Triclinic, $P\bar{1}$	Monoclinic, $P2_1/n$
Temperature (K)	293	293
a, b, c (Å)	9.9491 (17), 10.1521 (19), 10.5802 (18)	11.1105 (8), 8.7556 (6), 18.4838 (13)
α, β, γ (°)	63.440 (6), 67.862 (6), 86.962 (7)	90, 99.620 (3), 90
V (Å^3)	876.8 (3)	1772.8 (2)
Z	2	4
Radiation type	Mo $K\alpha$	
μ (mm^{-1})	1.30	8.45
Crystal size (mm)	0.13 × 0.07 × 0.06	0.55 × 0.36 × 0.12
Data collection		
Diffractometer	Bruker D8 VENTURE	
Absorption correction	Multi-scan *SADABS2016*/2 - Bruker AXS area detector scaling and absorption correction	
T_{min}, T_{max}	0.64, 0.93	0.17, 0.43
No. of measured, independent and observed [$I > 2\sigma(I)$] reflections	12156, 3269, 2519	26620, 3521, 2804

R_{int}	0.113	0.091
(sin θ/λ)$_{max}$ (Å^{-1})	0.609	0.620
Refinement		
$R[F^2 > 2\sigma(F^2)]$, $wR(F^2)$, S	0.097, 0.268, 1.06	0.032, 0.075, 1.03
No. of reflections	3269	3521
No. of parameters	211	201
H-atom treatment	H-atom parameters constrained	
$\Delta\rangle_{max}$, $\Delta\rangle_{min}$ (e Å^{-3})	3.18, -0.79	0.76, -0.85

Table S7. Stability of Cu-LA, Ag-LA and Au-LA in serum-free culture medium. Values indicate AUC, measured by HPLC-UV.

Cu-LA	Time							
measurement	0 h	1 h	2 h	4 h	8 h	16 h	32 h	48 h
1	1047	1022	1021	1040	1045	1039	1030	1038
2	1030	1016	1020	1041	1042	1042	1041	1039
3	1011	1021	1022	1043	1044	1042	1038	1040
4	1021	1017	1022	1044	1046	1046	1042	1040
5	1023	1017	1023	1044	1042	1042	1039	1039
**average**	**1026**	**1019**	**1022**	**1042**	**1044**	**1042**	**1038**	**1039**
Ag-LA	Time							
measurement	0 h	1 h	2 h	4 h	8 h	16 h	32 h	48 h
1	877	923	922	921	922	921	925	917
2	898	923	921	925	924	922	926	922
3	911	921	923	927	923	921	925	920
4	926	920	928	920	922	922	924	922
5	921	921	923	928	923	922	925	922
**average**	**907**	**922**	**923**	**924**	**923**	**922**	**925**	**921**
Au-LA	Time							
measurement	0 h	1 h	2 h	4 h	8 h	16 h	32 h	48 h
1	700	690	645	614	463	354	180	142
2	700	685	644	605	457	351	173	143
3	697	674	640	604	456	353	174	140
4	698	672	643	603	454	350	172	143
5	697	665	645	605	451	345	187	135
**average**	**698**	**677**	**643**	**606**	**456**	**351**	**177**	**141**

Figure S1.

Docking of estrone (yellow) and lepidiline A (pink) in the PDB structure 1QYV of human HSD17B1 in far view and close view, visualized by PyMOL. Surface has 20% transparency to see cartoon representation.

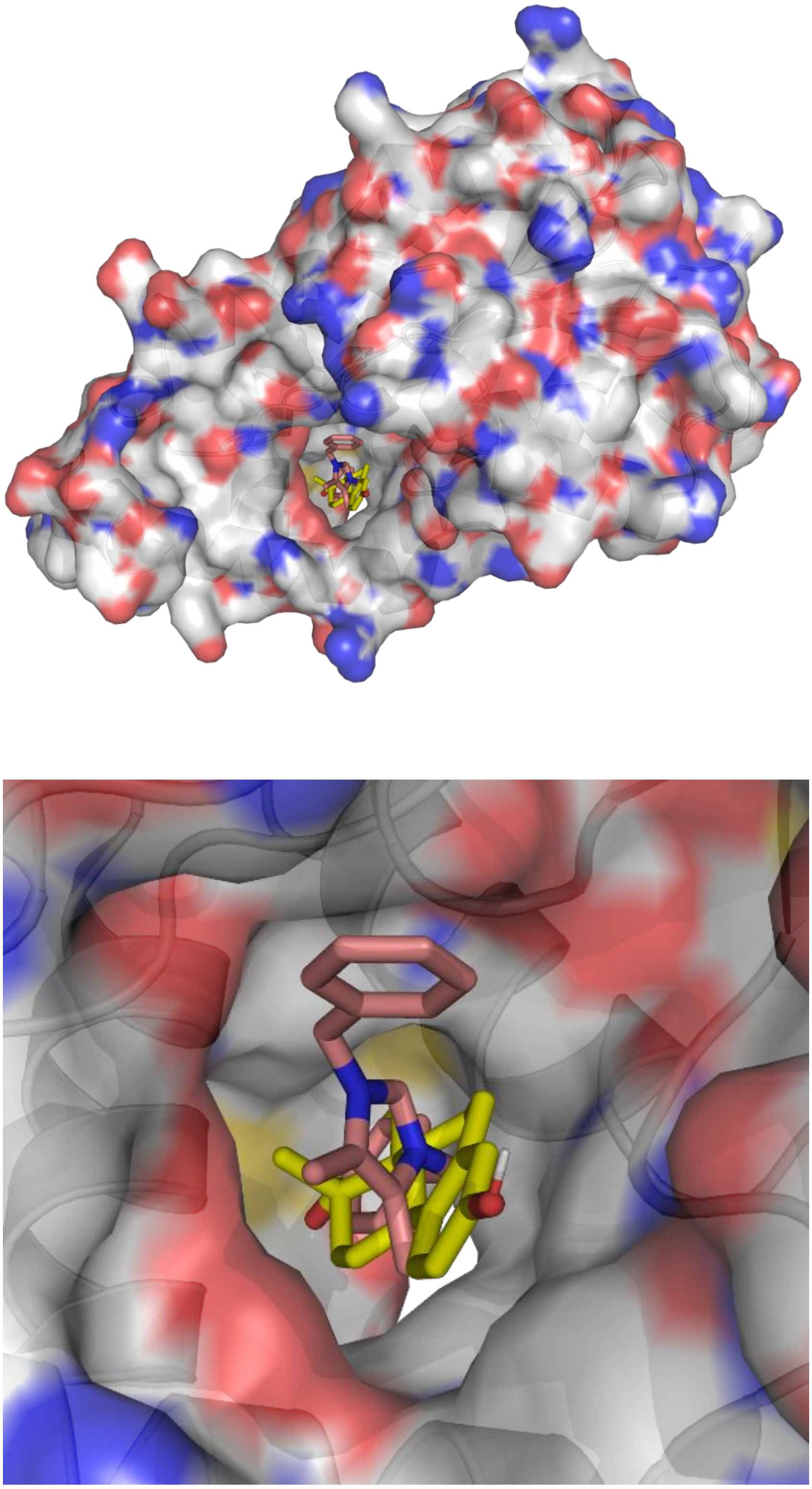

Figure S2. ^{1}H and ^{13}C NMR Spectra of 1,3-dibenzyl-4,5-dimethyl-imidazol-1-ium chloride (LA)

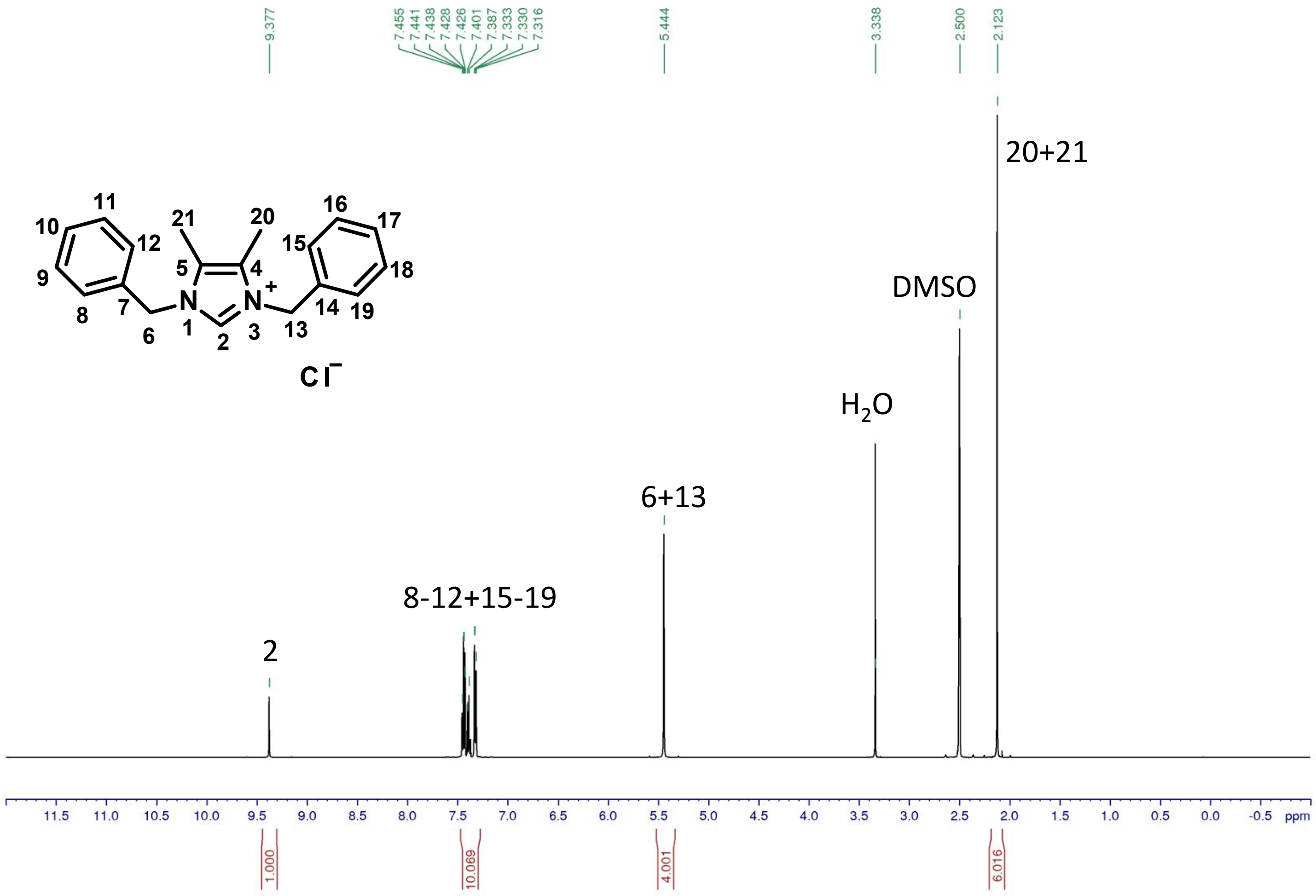

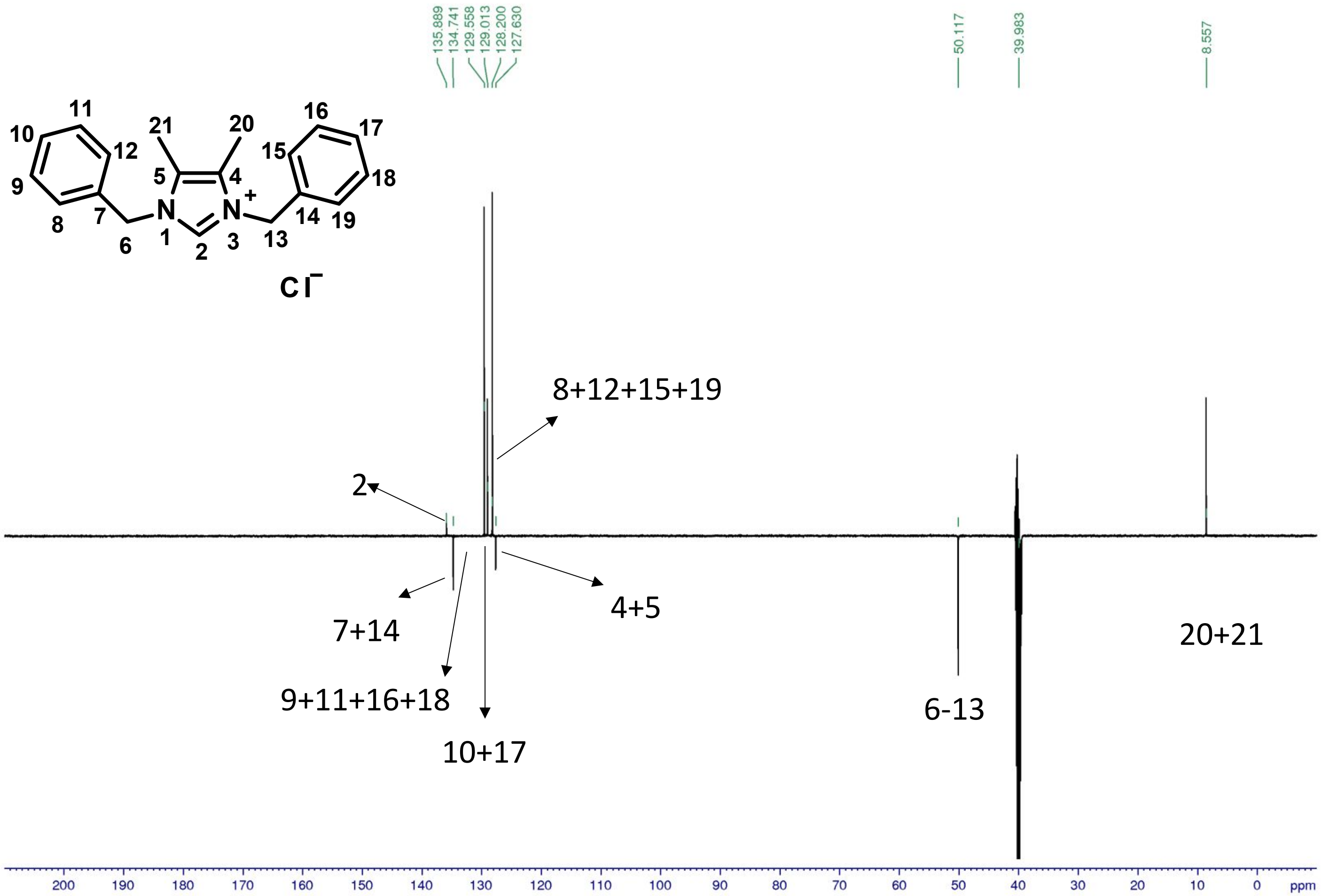

135.889
134.741
129.558
129.013
128.200
127.630
50.117
39.983
8.557
11
10
12
9
8
7
6
21
5
1
2
20
4
3
+
16
15
17
18
14
19
13
Cl⁻
8+12+15+19
2
7+14
9+11+16+18
10+17
4+5
6-13
20+21
200
190
180
170
160
150
140
130
120
110
100
90
80
70
60
50
40
30
20
10
0
ppm

Figure S3. ^1H and ^{13}C-NMR of chloro-(1,3-dibenzyl-4,5-dimethyl-imidazol-2-ylidene)copper (Cu-LA)

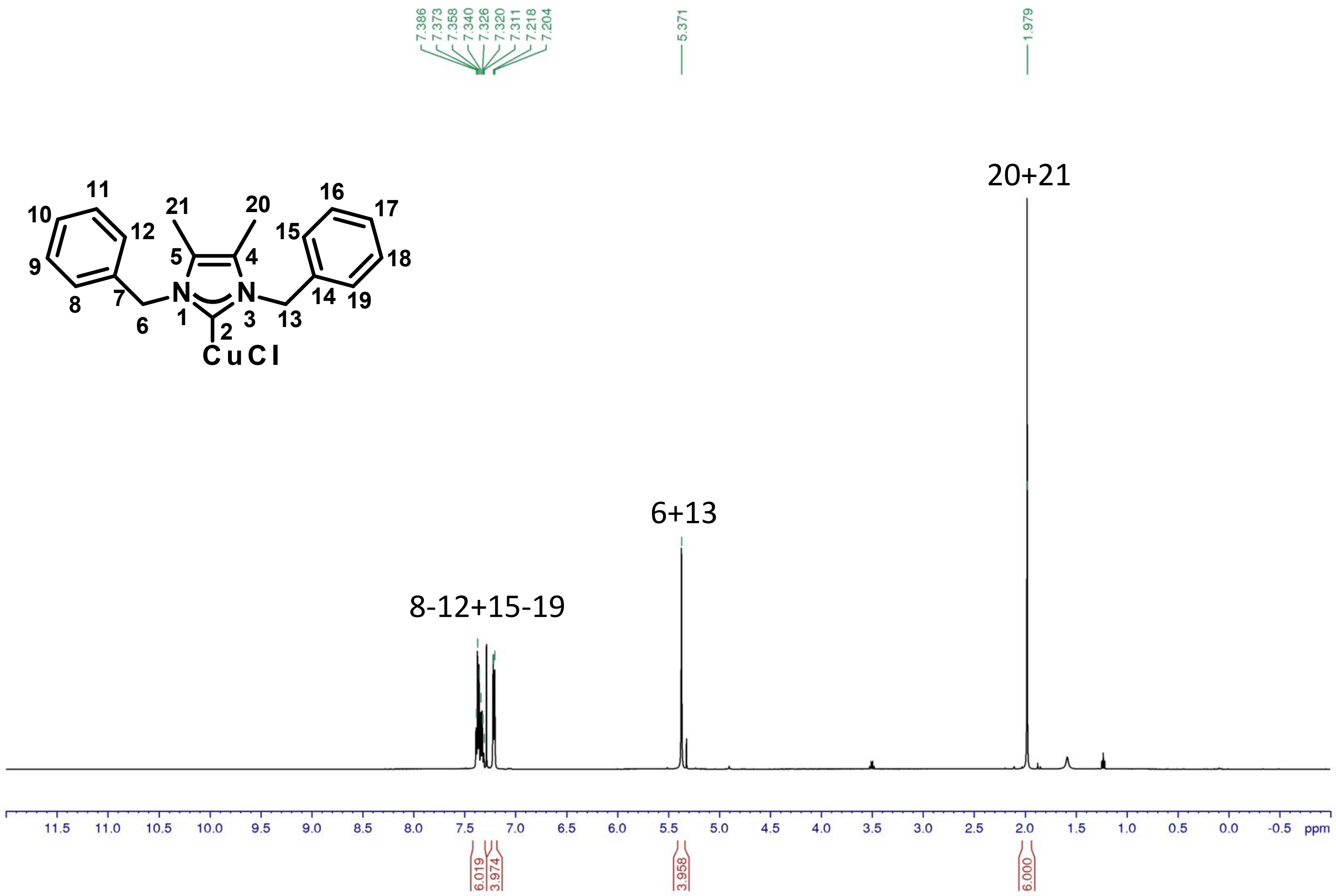

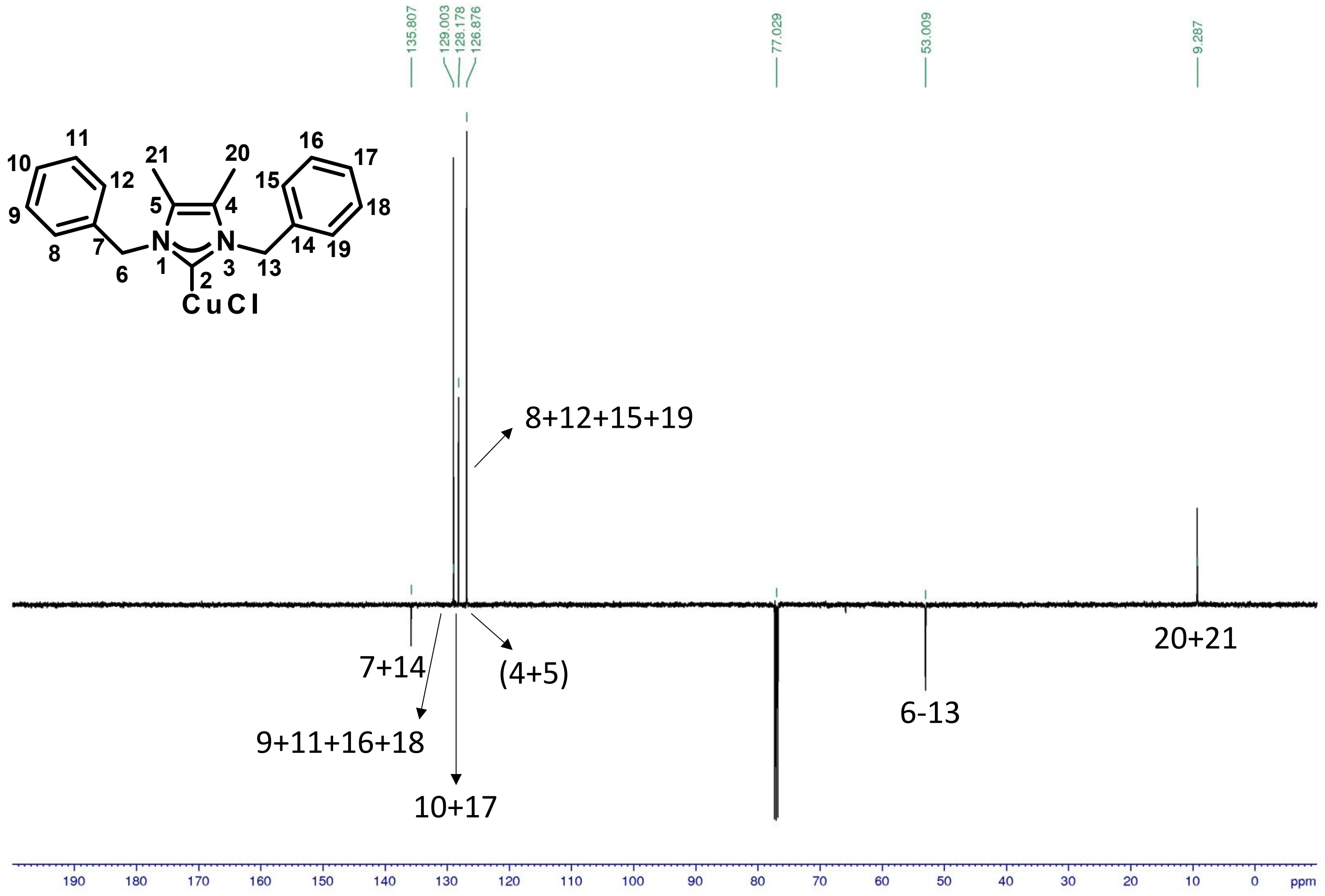

135.807
129.003
128.178
126.876
77.029
53.009
9.287
8+12+15+19
7+14
(4+5)
9+11+16+18
10+17
6-13
20+21
CuCl
190
180
170
160
150
140
130
120
110
100
90
80
70
60
50
40
30
20
10
0
ppm

Figure S4. ^{1}H and ^{13}C-NMR of chloro-(1,3-dibenzyl-4,5-dimethyl-imidazol-2-ylidene)silver (Ag-LA)

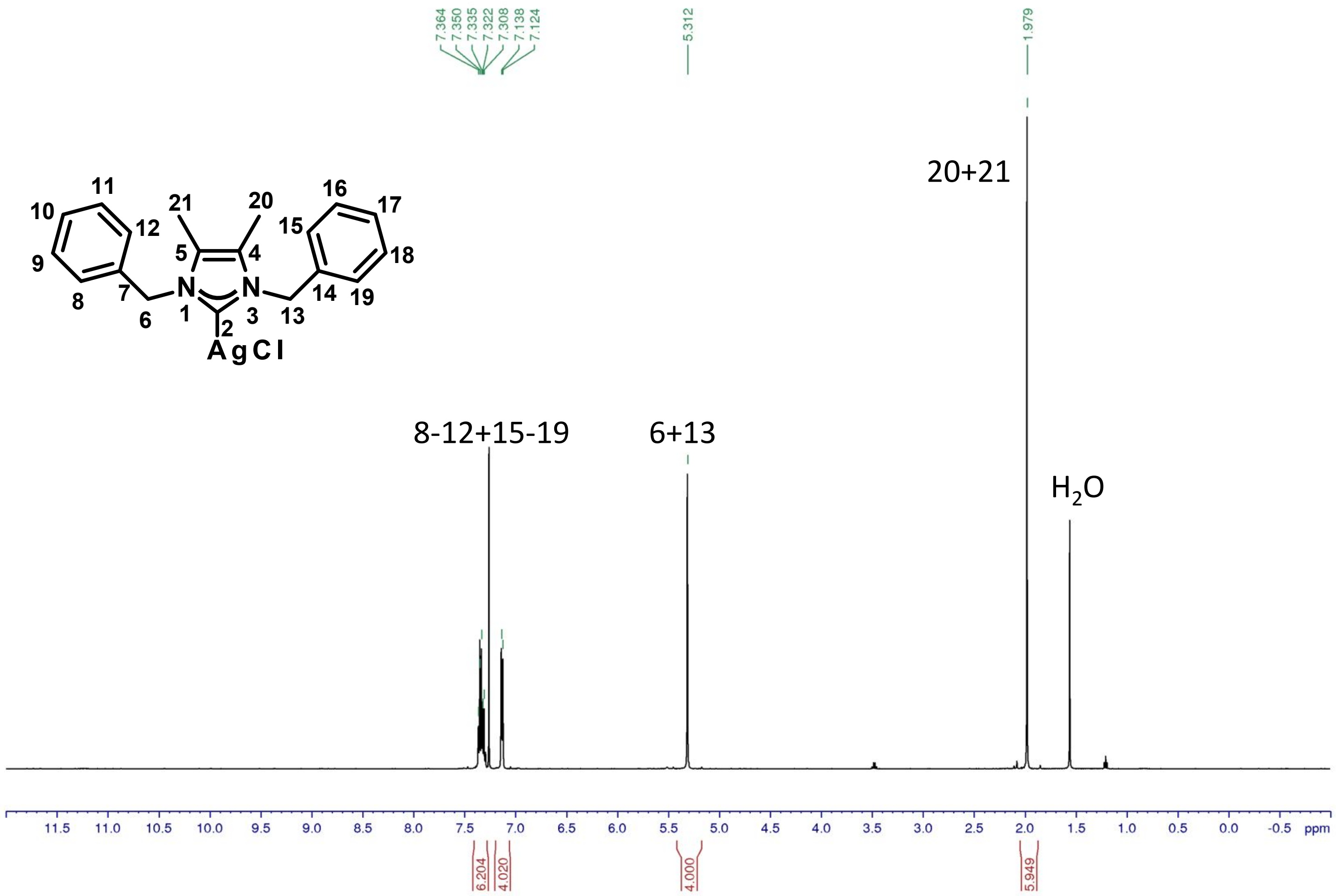

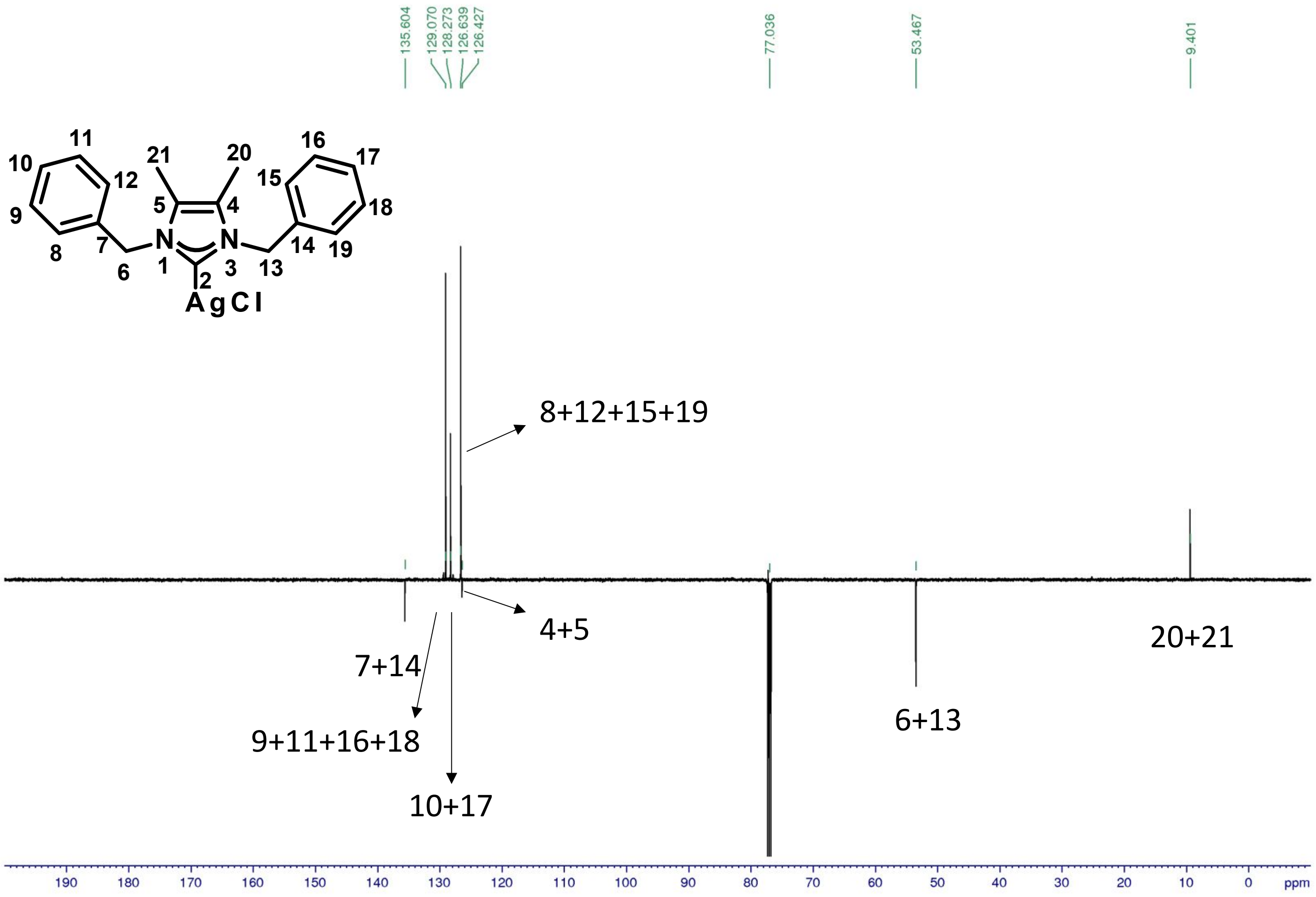

135.604
129.070
128.273
126.639
126.427
77.036
53.467
9.401
AgCl
8+12+15+19
4+5
7+14
9+11+16+18
10+17
6+13
20+21
190 180 170 160 150 140 130 120 110 100 90 80 70 60 50 40 30 20 10 0 ppm

Figure S5. ^{1}H and ^{13}C-NMR of chloro-(1,3-dibenzyl-4,5-dimethyl-imidazol-2-ylidene)gold (Au-LA)

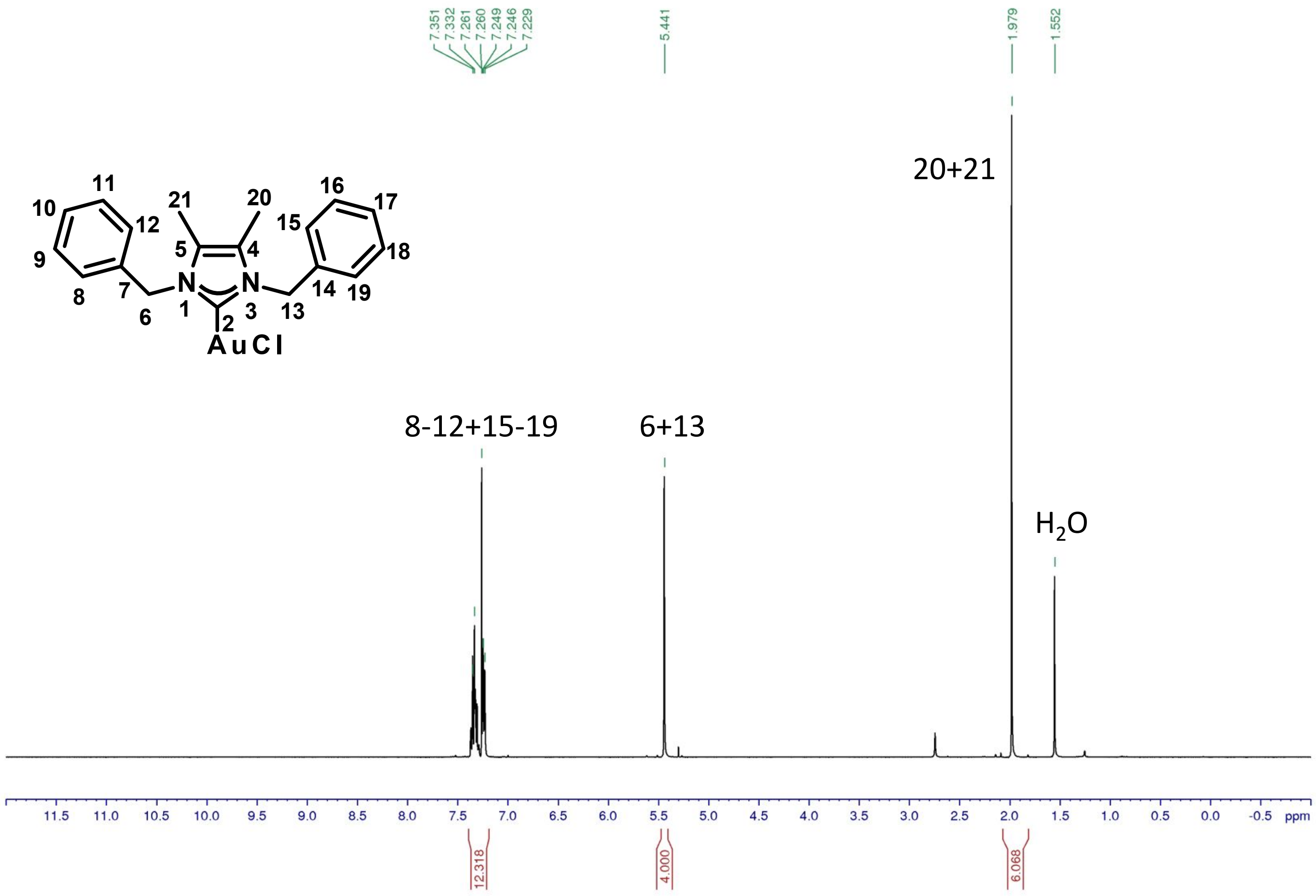

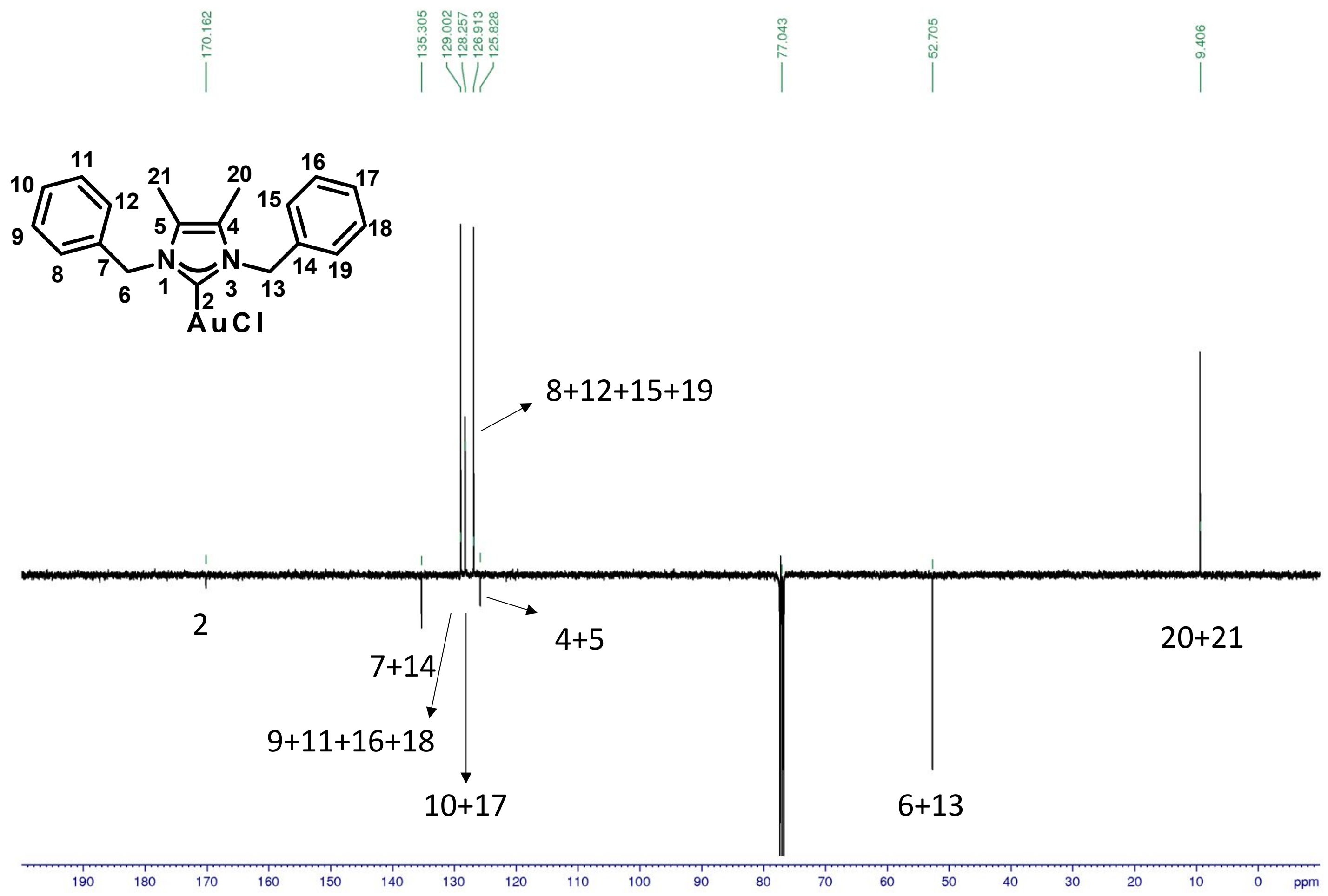

170.162
135.305
129.002
128.257
126.913
125.828
77.043
52.705
9.406
AuCl
8+12+15+19
2
7+14
9+11+16+18
10+17
4+5
6+13
20+21
190 180 170 160 150 140 130 120 110 100 90 80 70 60 50 40 30 20 10 0 ppm

Figure S6. X-ray structures and geometric parameters of Ag-LA. A) ORTEP view of Ag-LA complex at 50% probability level with partial numbering scheme. B) Packing diagram of Ag-LA structure with short contacts. Silver ions are connected through chloride bridges with a distance of 4.0 Å.

A) B)

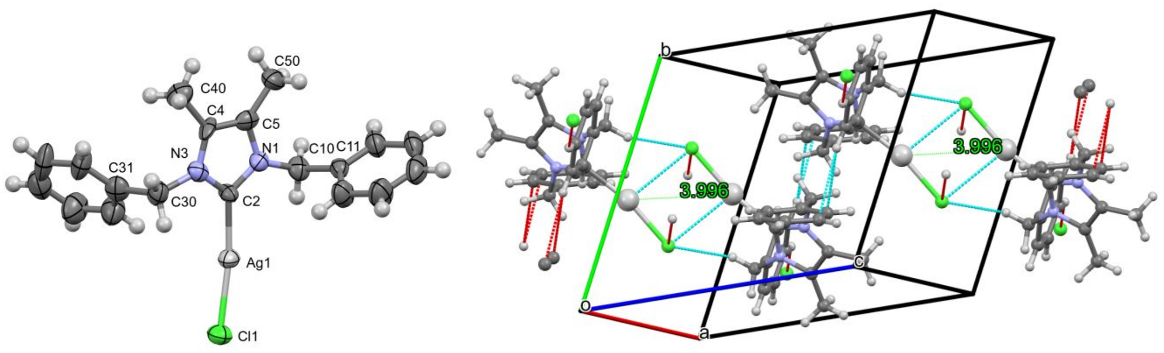

Geometric parameters of distances, angles and torsion angles (Å, º) for Ag-LA:

C2—N3	1.322 (13)	C30—N3	1.488 (15)
C2—N1	1.369 (13)	C30—C31	1.510 (19)
C2—Ag1	2.073 (9)	C30—H30A	0.9700
C4—N3	1.354 (14)	C30—H30B	0.9700
C4—C5	1.415 (18)	C31—C36	1.36 (2)
C4—C40	1.501 (16)	C31—C32	1.39 (2)
C5—N1	1.397 (14)	C32—C33	1.33 (2)
C5—C50	1.483 (17)	C32—H32	0.9300
C10—N1	1.445 (15)	C33—C34	1.35 (3)
C10—C11	1.501 (16)	C33—H33	0.9300
C10—H10A	0.9700	C34—C35	1.37 (2)
C10—H10B	0.9700	C34—H34	0.9300
C11—C16	1.374 (19)	C35—C36	1.36 (2)
C11—C12	1.389 (17)	C35—H35	0.9300
C12—C13	1.39 (2)	C36—H36	0.9300
C12—H12	0.9300	C40—H40A	0.9600
C13—C14	1.36 (2)	C40—H40B	0.9600
C13—H13	0.9300	C40—H40C	0.9600
C14—C15	1.38 (2)	C50—H50A	0.9600
C14—H14	0.9300	C50—H50B	0.9600
C15—C16	1.39 (2)	C50—H50C	0.9600
C15—H15	0.9300	Ag1—Cl1	2.337 (3)
C16—H16	0.9300		
Angles			
N3—C2—N1	103.9 (8)	H30A—C30—H30B	107.6000
N3—C2—Ag1	128.9 (7)	C36—C31—C32	116.7 (14)
N1—C2—Ag1	127.1 (7)	C36—C31—C30	121.9 (13)
N3—C4—C5	104.7 (9)	C32—C31—C30	121.3 (13)
N3—C4—C40	128.5 (11)	C33—C32—C31	121.8 (17)
C5—C4—C40	126.0 (11)	C33—C32—H32	119.1000
N1—C5—C4	104.7 (10)	C31—C32—H32	119.1000
N1—C5—C50	124.1 (12)	C32—C33—C34	121.2 (17)
C4—C5—C50	131.1 (11)	C32—C33—H33	119.4000
N1—C10—C11	112.6 (10)	C34—C33—H33	119.4000

N1—C10—H10A	109.1000	C33—C34—C35	118.5 (16)
C11—C10—H10A	109.1000	C33—C34—H34	120.8000
N1—C10—H10B	109.1000	C35—C34—H34	120.8000
C11—C10—H10B	109.1000	C36—C35—C34	120.5 (17)
H10A—C10—H10B	107.8000	C36—C35—H35	119.8000
C16—C11—C12	119.2 (12)	C34—C35—H35	119.8000
C16—C11—C10	122.0 (11)	C35—C36—C31	121.2 (16)
C12—C11—C10	118.7 (12)	C35—C36—H36	119.4000
C13—C12—C11	119.1 (15)	C31—C36—H36	119.4000
C13—C12—H12	120.5000	C4—C40—H40A	109.5000
C11—C12—H12	120.5000	C4—C40—H40B	109.5000
C14—C13—C12	121.4 (13)	H40A—C40—H40B	109.5000
C14—C13—H13	119.3000	C4—C40—H40C	109.5000
C12—C13—H13	119.3000	H40A—C40—H40C	109.5000
C13—C14—C15	120.0 (14)	H40B—C40—H40C	109.5000
C13—C14—H14	120.0000	C5—C50—H50A	109.5000
C15—C14—H14	120.0000	C5—C50—H50B	109.5000
C14—C15—C16	119.1 (16)	H50A—C50—H50B	109.5000
C14—C15—H15	120.5000	C5—C50—H50C	109.5000
C16—C15—H15	120.5000	H50A—C50—H50C	109.5000
C11—C16—C15	121.1 (14)	H50B—C50—H50C	109.5000
C11—C16—H16	119.4000	C2—Ag1—Cl1	167.7 (3)
C15—C16—H16	119.4000	C2—N1—C5	111.3 (9)
N3—C30—C31	114.8 (11)	C2—N1—C10	125.2 (9)
N3—C30—H30A	108.6000	C5—N1—C10	123.5 (10)
C31—C30—H30A	108.6000	C2—N3—C4	115.0 (9)
N3—C30—H30B	108.6000	C2—N3—C30	122.8 (9)
C31—C30—H30B	108.6000	C4—N3—C30	122.0 (10)
Torsion angles			
N3—C4—C5—N1	-6.2 (12)	C32—C31—C36—C35	-2 (3)
C40—C4—C5—N1	-177.0 (12)	C30—C31—C36—C35	174.6 (16)
N3—C4—C5—C50	178.5 (12)	N3—C2—N1—C5	0.2 (12)
C40—C4—C5—C50	8 (2)	Ag1—C2—N1—C5	176.8 (8)
N1—C10—C11—C16	51.2 (16)	N3—C2—N1—C10	-178.7 (10)
N1—C10—C11—C12	-133.5 (12)	Ag1—C2—N1—C10	-2.1 (16)
C16—C11—C12—C13	-2.1 (19)	C4—C5—N1—C2	3.9 (12)
C10—C11—C12—C13	-177.6 (12)	C50—C5—N1—C2	179.6 (11)
C11—C12—C13—C14	1 (2)	C4—C5—N1—C10	-177.2 (10)
C12—C13—C14—C15	-1 (2)	C50—C5—N1—C10	-1.5 (17)
C13—C14—C15—C16	3 (3)	C11—C10—N1—C2	-106.9 (12)
C12—C11—C16—C15	4 (2)	C11—C10—N1—C5	74.3 (14)
C10—C11—C16—C15	179.5 (14)	N1—C2—N3—C4	-4.6 (13)
C14—C15—C16—C11	-5 (3)	Ag1—C2—N3—C4	178.8 (9)
N3—C30—C31—C36	70.6 (18)	N1—C2—N3—C30	-179.2 (10)
N3—C30—C31—C32	-113.0 (14)	Ag1—C2—N3—C30	4.3 (16)
C36—C31—C32—C33	1 (2)	C5—C4—N3—C2	7.0 (14)
C30—C31—C32—C33	-175.9 (15)	C40—C4—N3—C2	177.5 (13)
C31—C32—C33—C34	2 (3)	C5—C4—N3—C30	-178.4 (10)
C32—C33—C34—C35	-3 (3)	C40—C4—N3—C30	-8 (2)
C33—C34—C35—C36	2 (3)	C31—C30—N3—C2	-97.4 (14)
C34—C35—C36—C31	1 (3)	C31—C30—N3—C4	88.4 (14)

Figure S7. X-ray structures and geometric parameters of Au-LA. A) ORTEP view of Au-LA complex at 50% probability level with partial numbering scheme. C31 and C14 are refined isotropically. B) Packing diagram of Au-LA with 3.9 Å of gold-gold distance, and 5.2 Å distance of Au-Cl with the neighboring molecule, indicating that the chloride ion is not bridging. The dimers are connected by C-H..Cl hydrogen bonds through a phenyl proton with H..Cl distance of 2.9 Å.

A)

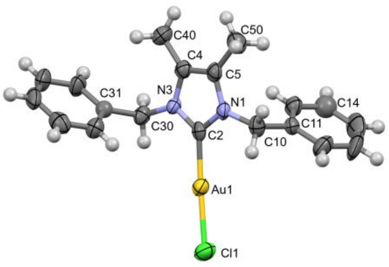

B)

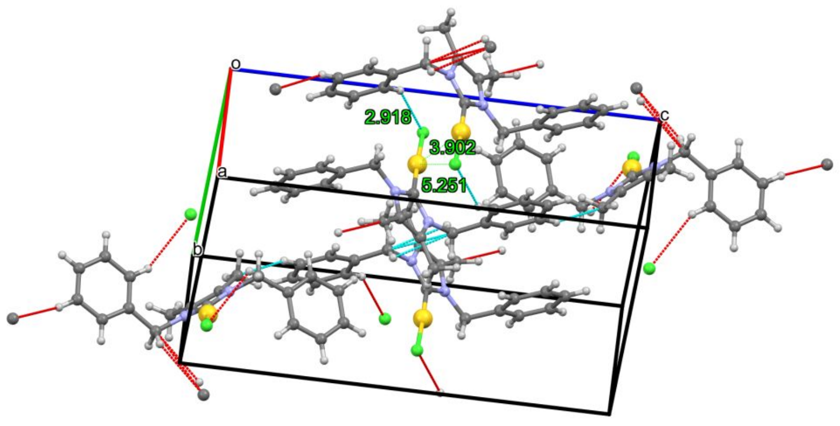

Geometric parameters of distances, angles and torsion angles (Å, º) for Au-LA:

C2—N1	1.343 (7)	C30—N3	1.470 (7)
C2—N3	1.345 (7)	C30—C31	1.499 (8)
C2—Au1	1.979 (6)	C30—H30A	0.9700
C4—C5	1.352 (8)	C30—H30B	0.9700
C4—N3	1.385 (7)	C31—C32	1.376 (8)
C4—C40	1.476 (9)	C31—C36	1.379 (8)
C5—N1	1.385 (8)	C32—C33	1.368 (9)
C5—C50	1.487 (8)	C32—H32	0.9300
C10—N1	1.449 (8)	C33—C34	1.382 (11)
C10—C11	1.517 (8)	C33—H33	0.9300
C10—H10A	0.9700	C34—C35	1.378 (11)
C10—H10B	0.9700	C34—H34	0.9300
C11—C12	1.368 (9)	C35—C36	1.380 (9)
C11—C16	1.370 (8)	C35—H35	0.9300
C12—C13	1.368 (12)	C36—H36	0.9300
C12—H12	0.9300	C40—H40A	0.9600
C13—C15	1.347 (13)	C40—H40B	0.9600
C13—H13	0.9300	C40—H40C	0.9600
C14—C15	1.349 (12)	C50—H50A	0.9600
C14—C16	1.370 (9)	C50—H50B	0.9600
C14—H14	0.9300	C50—H50C	0.9600
C15—H15	0.9300	Cl1—Au1	2.2787 (17)
C16—H16	0.9300		
N1—C2—N3	104.7 (5)	H30A—C30—H30B	107.7000
N1—C2—Au1	125.2 (4)	C32—C31—C36	118.7 (6)
N3—C2—Au1	130.1 (4)	C32—C31—C30	121.2 (5)
C5—C4—N3	106.9 (5)	C36—C31—C30	120.1 (5)
C5—C4—C40	130.0 (6)	C33—C32—C31	121.0 (7)
N3—C4—C40	123.1 (5)	C33—C32—H32	119.5000
C4—C5—N1	105.7 (5)	C31—C32—H32	119.5000

C4—C5—C50	130.5 (6)	C32—C33—C34	120.7 (7)
N1—C5—C50	123.8 (6)	C32—C33—H33	119.7000
N1—C10—C11	114.1 (5)	C34—C33—H33	119.7000
N1—C10—H10A	108.7000	C35—C34—C33	118.5 (7)
C11—C10—H10A	108.7000	C35—C34—H34	120.7000
N1—C10—H10B	108.7000	C33—C34—H34	120.7000
C11—C10—H10B	108.7000	C34—C35—C36	120.8 (7)
H10A—C10—H10B	107.6000	C34—C35—H35	119.6000
C12—C11—C16	118.5 (6)	C36—C35—H35	119.6000
C12—C11—C10	118.7 (6)	C31—C36—C35	120.4 (6)
C16—C11—C10	122.8 (5)	C31—C36—H36	119.8000
C13—C12—C11	120.1 (8)	C35—C36—H36	119.8000
C13—C12—H12	119.9000	C4—C40—H40A	109.5000
C11—C12—H12	119.9000	C4—C40—H40B	109.5000
C15—C13—C12	120.0 (8)	H40A—C40—H40B	109.5000
C15—C13—H13	120.0000	C4—C40—H40C	109.5000
C12—C13—H13	120.0000	H40A—C40—H40C	109.5000
C15—C14—C16	118.8 (8)	H40B—C40—H40C	109.5000
C15—C14—H14	120.6000	C5—C50—H50A	109.5000
C16—C14—H14	120.6000	C5—C50—H50B	109.5000
C13—C15—C14	121.3 (8)	H50A—C50—H50B	109.5000
C13—C15—H15	119.3000	C5—C50—H50C	109.5000
C14—C15—H15	119.3000	H50A—C50—H50C	109.5000
C14—C16—C11	121.2 (7)	H50B—C50—H50C	109.5000
C14—C16—H16	119.4000	C2—N1—C5	111.8 (5)
C11—C16—H16	119.4000	C2—N1—C10	123.8 (5)
N3—C30—C31	113.3 (4)	C5—N1—C10	124.2 (5)
N3—C30—H30A	108.9000	C2—N3—C4	110.9 (5)
C31—C30—H30A	108.9000	C2—N3—C30	124.4 (5)
N3—C30—H30B	108.9000	C4—N3—C30	124.7 (5)
C31—C30—H30B	108.9000	C2—Au1—Cl1	176.82 (17)
N3—C4—C5—N1	0.7 (6)	C30—C31—C36—C35	-179.3 (6)
C40—C4—C5—N1	-176.5 (6)	C34—C35—C36—C31	-0.1 (10)
N3—C4—C5—C50	179.8 (6)	N3—C2—N1—C5	-0.4 (6)
C40—C4—C5—C50	2.6 (11)	Au1—C2—N1—C5	-178.9 (4)
N1—C10—C11—C12	-168.4 (6)	N3—C2—N1—C10	-175.2 (5)
N1—C10—C11—C16	12.4 (8)	Au1—C2—N1—C10	6.3 (7)
C16—C11—C12—C13	0.9 (10)	C4—C5—N1—C2	-0.2 (6)
C10—C11—C12—C13	-178.3 (7)	C50—C5—N1—C2	-179.4 (5)
C11—C12—C13—C15	-1.6 (13)	C4—C5—N1—C10	174.6 (5)
C12—C13—C15—C14	1.2 (15)	C50—C5—N1—C10	-4.6 (8)
C16—C14—C15—C13	-0.2 (13)	C11—C10—N1—C2	88.5 (6)
C15—C14—C16—C11	-0.5 (11)	C11—C10—N1—C5	-85.7 (7)
C12—C11—C16—C14	0.1 (10)	N1—C2—N3—C4	0.9 (6)
C10—C11—C16—C14	179.3 (6)	Au1—C2—N3—C4	179.3 (4)
N3—C30—C31—C32	122.9 (6)	N1—C2—N3—C30	179.0 (4)
N3—C30—C31—C36	-59.0 (7)	Au1—C2—N3—C30	-2.6 (7)
C36—C31—C32—C33	1.4 (10)	C5—C4—N3—C2	-1.0 (6)
C30—C31—C32—C33	179.5 (6)	C40—C4—N3—C2	176.5 (5)
C31—C32—C33—C34	-0.4 (11)	C5—C4—N3—C30	-179.1 (5)
C32—C33—C34—C35	-0.9 (11)	C40—C4—N3—C30	-1.7 (8)
C33—C34—C35—C36	1.1 (11)	C31—C30—N3—C2	113.7 (6)
C32—C31—C36—C35	-1.2 (9)	C31—C30—N3—C4	-68.4 (7)
